# PrP^C^ Glycoprotein Is Indispensable for Maintenance of Skeletal Muscle Homeostasis During Aging

**DOI:** 10.1002/jcsm.13706

**Published:** 2025-01-28

**Authors:** Wenduo Liu, Thi Thu Trang Kieu, Zilin Wang, Hyun‐Jaung Sim, Seohyeong Lee, Jeong‐Chae Lee, Yoonjung Park, Sang Hyun Kim, Sung‐Ho Kook

**Affiliations:** ^1^ Department of Sports Science, College of Natural Science Jeonbuk National University Jeonju Republic of Korea; ^2^ Department of Bioactive Material Sciences, Research Center of Bioactive Materials Jeonbuk National University Jeonju Republic of Korea; ^3^ Cluster for Craniofacial Development and Regeneration Research, Institute of Oral Biosciences and School of Dentistry Jeonbuk National University Jeonju Republic of Korea; ^4^ Department of Nutritional Sciences University of California Berkeley Berkeley California USA; ^5^ Laboratory of Integrated Physiology, Department of Health & Human Performance University of Houston Texas USA

**Keywords:** ER stress, mitochondria damage, PrP^C^, satellite stem cell senescence, skeletal muscle homeostasis

## Abstract

**Background:**

The cellular prion protein (PrP^C^), a glycoprotein encoded by the *PRNP* gene, is known to modulate muscle mass and exercise capacity. However, the role of PrP^C^ in the maintenance and regeneration of skeletal muscle during ageing remains unclear.

**Methods:**

This study investigated the change in PrP^C^ expression during muscle formation using C2C12 cells and evaluated muscle function in *Prnp* wild‐type (WT) and knock‐out (KO) mice at different ages (1, 9 and 15 months). To determine the role of PrP^C^ in skeletal muscle homeostasis during ageing, we conducted regeneration experiments via cardiotoxin injection in *Prnp* mice to assess the effects of PrP^C^ deficiency on the senescence of satellite stem cells (SCs) and regenerative capacity in skeletal muscle.

**Results:**

Our data demonstrate that PrP^C^ expression increased significantly during muscle differentiation (*p* < 0.01), correlating with myogenin (immunofluorescence at the differentiation stage). PrP^C^ deficiency disrupted muscle homeostasis, leading to age‐associated mitochondrial autophagy (Pink‐1, +180%, *p* < 0.001; Parkin, +161%, *p* < 0.01) and endoplasmic reticulum stress (SERCA, −26%, *p* < 0.05; IRE1*α*, +195%, *p* < 0.001) while decreasing the level of mitochondrial biogenesis (SIRT‐1, −50%, *p* < 0.01; PGC‐1*α*, −36%, *p* < 0.05; VDAC, −27%, *p* < 0.001), and activated oxidative stress (serum myoglobin, +23%, *p* < 0.001; MDA, +23%, *p* < 0.05; NF*κ*B, +117%, p < 0.05) during ageing, which accelerated reduced muscle growth or mass accumulation (tibialis anterior muscle mass, −23%, *p* < 0.001; gastrocnemius muscle mass, −30%, *p* < 0.001; muscle fibre size, −48%, *p* < 0.05; MSTN, +160%, *p* < 0.01; MAFbx, +83%, *p* < 0.05). Furthermore, PrP^C^ deficiency induced the senescence (*β*‐galactosidase, +60%, *p* < 0.05; p16, +103%, *p* < 0.001) of SCs, which was directly related to the defect in muscle recovery, with the senescence‐mediated enhancement of adipogenesis (PPAR*γ*, +74%, *p* < 0.05) during the regeneration process after cardiotoxin‐induced muscle injury.

**Conclusions:**

Our findings demonstrate that PrP^C^ is indispensable for maintaining skeletal muscle homeostasis during ageing by modulating the functional integrity of mitochondria, ER and SCs.

## 1 Introduction

The decline in skeletal muscle mass and function with ageing, known as sarcopenia, is a critical issue due to the essential role of skeletal muscles in facilitating controlled movement [1], influencing metabolism [2] and supporting overall bodily functions and health [3, 4]. Ageing leads to alteration in skeletal muscle mitochondria morphology, quality and quantity, resulting in mitochondrial function characterized by decreased activity of respiratory complexes, downregulation of oxidative phosphorylation and mitochondrial protein homeostasis genes [5, 6]. This decline in muscle mass and function contributes to limitations in daily activities, such as reduced strength and impaired mitochondrial function, potentially leading to metabolic dysfunction and various age‐related conditions [5, 7], ultimately compromising the health of older individuals. Sarcopenia is intricately linked to processes such as muscle regeneration [8], cellular senescence [9], apoptosis [10], oxidative stress [11] and inflammation [12, 13] and the deterioration of stem cell function [14, 15], further exacerbating the loss of muscle mass and regeneration capacity [14]. Therefore, understanding the underlying mechanisms of sarcopenia and identifying potential therapeutic targets is crucial for mitigating age‐related declines in muscle function and improving the quality of life for older adults.

The ability to maintain skeletal muscle homeostasis is pivotal for skeletal muscle health and quality of life during ageing [16]. PrP^C^ glycoprotein, encoded by the *PRNP* gene, is essential for skeletal muscle function. However, both overexpression and deficiency of PrP^C^ can threaten skeletal muscle health by affecting the differentiation of adult myoblasts via different mechanisms [17, 18, 19]. Overexpression of PrP^C^ has been shown to induce primary myopathy [17], while excessive PrP^C^ accumulation inhibits autophagy in skeletal muscle cells, impairing myoblast differentiation, and affecting regenerative capacity [18]. In contrast, a lack of PrP^C^ negatively impacts muscle mass and exercise capacity. Several studies report no significant differences in treadmill performance between young *Prnp* KO and their WT counterparts although the performance declines significantly with age [20, 21]. Additionally, two‐month‐old *Prnp* KO mice exhibit impaired locomotor performance under more extreme exercise conditions, potentially linked to mitochondrial abnormalities [22]. PrP^C^ expression is associated with cellular regulation of oxidative stress in skeletal muscle [23]. Interestingly, PrP^C^ levels vary according to skeletal muscle fibre type and are critical for maintaining normal redox homeostasis, muscle size and contractile function in adult animals [24]. However, the regulatory mechanism by which PrP^C^ regulates oxidative and antioxidant functions in skeletal muscle remains unclear. Moreover, PrP^C^ dysfunction may prevent normal differentiation of myotubes [25], and in atrophic muscles, PrP^C^ expression is mainly restricted to regenerating fibres [26], suggesting a potential regulatory role of PrP^C^ glycoprotein in skeletal muscle development and regeneration. Despite the recognition of PrP^C^'s roles in various skeletal muscle functions, its role and mechanisms for maintaining and regenerating skeletal muscle during ageing remain poorly understood. In this current study, we thus determined the role of PrP^C^ in regulating skeletal muscle maintenance and regeneration during ageing by using *Prnp* KO mice of different ages and cardiotoxin‐induced muscle regeneration model.

In contrast to previous studies, the present study focused more on the role of PrP^C^ in maintaining skeletal muscle function and homeostasis during age‐related development, as well as the progressive decline in muscle health in the absence of PrP^C^. Ultimately, this study aims to provide a clearer understanding of PrP^C^'s involvement in the regulation of skeletal muscle systems, particularly in the context of ageing and regeneration.

## 2 Methods

### 2.1 Animals

*Prnp* (encoding PrP^C^) KO mice on the FVB background were purchased from The Jackson Laboratory (Bar Harbour, ME, USA), as detailed in a previous publication [27]. The mice were housed under controlled laboratory conditions, with 18°C–22°C temperature, 40%–60% humidity, and a 12‐h light–dark cycle (08:00–20:00). This study was approved by the Institutional Animal Care and Use Committee of Jeonbuk National University (IACUC approval no. CBNU‐2022‐0067).

### 2.2 Genotyping of Prnp Knockout Mice

The polymerase chain reaction (PCR) mixture included 2.5 μL of 10 × H‐star Taq buffer, 5 μL of 5 × band helper, 1 μL of 10 mM dNTP mix, 1 μL each of primer (10 μM), 0.2 μL of H‐star Taq DNA polymerase (BIOFACT, Korea) and nuclease‐free water to 25 μL. PCR conditions: 95°C for 15 min; 34 cycles of 95°C for 20 s, 56°C for 40 s, 72°C for 1 min; final extension at 72°C for 5 min. PCR was run on a C1000 Touch Thermal Cycler (Bio‐Rad, USA). Products were stained with ethidium bromide, separated on 1% agarose gels, and genotyped (wild‐type: 388 bp; mutant: 341 bp). Sequencing was done on an ABI 3730 sequencer (Applied Biosystems, USA) and confirmed with Finch TV (Geospiza, USA).

### 2.3 Injection of Cardiotoxin Into Skeletal Muscle

The 50 μL (10 μM) cardiotoxin (CTX; Latoxan, Valence, France) was injected into the tibialis anterior (TA) muscle of four‐month‐old wild‐type (WT) and knock‐out (KO) mice [28]. Then samples were collected at 7 or 28 post‐injection to assess skeletal muscle regeneration [29].

### 2.4 Endurance Capacity Test

The endurance capacity test was conducted on a treadmill with a 15° slope. Treadmill speed started at 10 m/min for 5 min, then increased by 2 m/min every minute. The test was terminated when a mouse stayed on the shock grid for over 3 s despite sponge stimulation. The total running time, vertical distance, and work were measured or calculated as previously described [30].

### 2.5 Grip Strength Test

The peak grip strength of mice forelimbs and hindlimbs was measured using a grip strength metre (Ugo Basile, Cat.No.47200, Italy). For each test, the mouse grips the bar/net, and tension on the tail increases until detachment, recording the maximum grip force. Each mouse underwent 5 trials to ensure reliable measurements [31].

### 2.6 Oral Glucose Tolerance Test

After a 14‐h fast, mice received a gavage of 20% glucose solution (Sigma‐Aldrich, 158 968, USA; 2‐g glucose per kg body weight). Blood glucose was measured from tail tip samples at 0, 15, 30, 60, 90 and 120 min post‐gavage using a glucometer (Roche, ACCU‐CHEK, Germany). The blood glucose change curve and area under the curve were calculated after test completion [32, 33].

### 2.7 Tissue Weight Analysis

Bilateral TA and gastrocnemius (Gas) muscles were extracted for tissue weight analysis using an electronic balance. After measurement, muscles were either formalin‐fixed or frozen at −80°C.

### 2.8 Dual‐Energy X‐Ray Absorptiometry Analysis (DXA)

Anaesthetised mice underwent DXA scanning (iNSiGHT VET DXA, South Korea) to measure lean body weight and bone mineral density (BMD) by labelling whole mice as regions of interest (ROI).

### 2.9 C2C12 Cell Culture and Differentiation

C2C12 myoblasts (American Type Culture Collection, ATCC, USA) were cultured in Dulbecco's modified Eagle's medium (DMEM) supplemented with 10% foetal bovine serum (FBS) and 1% antibiotics (growth medium; GM) at 37°C. Upon reaching 80% confluence, myogenic differentiation was induced using DMEM supplemented with 2% horse serum (differentiation medium; DM) and cultured under 5% CO_2_/95% air at 37°C [34].

### 2.10 Biological Transmission Electron Microscopy Analysis

Transmission electron microscopy (TEM) was used to examine mitochondrial structure in the extensor digitorum longus (EDL) muscle. The muscle was fixed with 2.5% glutaraldehyde and 4% formaldehyde in 0.1 M phosphate buffer (pH 7.4) for 2 h, followed by post‐fixation with 1% osmium tetroxide for 2 h. After dehydration in a graded ethanol series, the muscle was embedded in Epon‐812 resin. Thin sections (~80 nm) were cut using a NOVA ultramicrotome (LKB, Vienna, Austria) and mounted on a 100‐mesh grid. Sections were stained with uranyl acetate and lead citrate and examined with an electron microscope (H7650, 80 kV, Hitachi, Japan). TEM analysis was performed using a JEM‐2010 microscope (JEOL) at the Center for University‐Wide Research Facilities (CURF) at Jeonbuk National University.

### 2.11 H&E Staining Analysis

TA muscle samples was performed according to a previously developed protocol [35]. Tissues were fixed, embedded, sectioned, stained with H&E, scanned using a Motic Easy Scan One slide scanner (Meyer Instruments, Inc., Houston, TX, USA) and analysed with ImageJ software (Version 1.51, NIH, Bethesda, MD, USA) to quantify muscle fibre size.

### 2.12 Immunofluorescence and Confocal Microscopy Analysis

Fixed C2C12 cells and rehydrated TA muscle sections were permeabilized, blocked, and incubated with primary antibodies: VDAC‐1 (Abcam, ab15895, USA); Parkin (SCBT, sc‐32 282, USA); PRNP (Cell Signaling, #14025, USA); *β*‐Galactosidase (Cell Signaling, #27198, USA); P16 (SCBT, sc‐759, USA); Pax‐7 (SCBT, sc‐81 648, USA); MyoD (SCBT, sc‐377 460, USA); Myogenin (SCBT, sc‐52 903, USA). After incubation with secondary antibodies (Anti‐Rabbit Alexa Fluor 488, ab150077; Goat Anti‐Mouse Alexa Fluor 594, ab150116, USA), samples were mounted with DAPI‐containing mounting medium (ChemCruz, sc‐24 941, USA) and analysed using a super‐resolution laser confocal scanning microscope.

### 2.13 TBARS Assay

Malondialdehyde (MDA) concentration in skeletal muscle protein extracts was measured using a TBARS assay kit (Cayman, No. 10009055, USA). Samples were processed, incubated and absorbance at 532 nm was read to calculate results [36].

### 2.14 Enzyme‐Linked Immunosorbent Assay

Blood samples were collected from mouse hearts and allowed to clot for 2 h at room temperature. The samples were then centrifuged at 3000 rpm for 15 min to collect the upper serum layer. Measurement of serum myoglobin levels was conducted using an enzyme‐linked immunosorbent assay (ELISA) kit (MyBioSource, MBS724191, California, USA).

### 2.15 Western Blot Analysis

Gas muscle extracts were prepared, and western blotting was performed as described previously [36]. The antibodies used are as follows: *β*‐actin (Invitrogen, MA1–140, USA); PrP^C^ (SPI‐bio, SAF32, France); VDAC‐1 (Abcam, ab15895, USA); PINK‐1 (SCBT, sc‐517 353, USA); Parkin (SCBT, sc‐32 282, USA); SERCA (SCBT, sc‐271 669, USA); IRE1*α* (SCBT, sc‐390 960, USA); PGC‐1*α* (Gene Tex, GTX37356, USA); SIRT‐1 (Millipore, #07–131, USA); OXPHOS (Abcam, ab110413, USA); GLUT‐4 (SCBT, sc‐53 566, USA); LDH (SCBT, sc‐133 123, USA); HXK‐2 (SCBT, sc‐6521, USA); PFK‐1 (SCBT, sc‐166 722, USA); NFκB (SCBT, sc‐8008, USA); Catalase (SCBT, sc‐271 358, USA); GPx‐4 (SCBT, sc‐166 570, USA); Pax‐7 (SCBT, sc‐81 648, USA); MyoD (SCBT, sc‐377 460, USA); Myogenin (SCBT, sc‐52 903, USA); PI3K (SCBT, sc‐1637, USA); P70S6K(SCBT, sc‐8418, USA); MuRF‐1 (SCBT, sc‐398 608, USA); MAFbx (SCBT, sc‐166 806, USA); Myostatin (SCBT, sc‐134 345, USA); PPAR*γ* (SCBT, sc‐7273, USA); mouse anti‐goat (SCBT, sc‐2354, USA), mouse anti‐rabbit (SCBT, sc‐2357, USA) or goat anti‐mouse (SCBT, sc‐2005, USA).

### 2.16 Statistical Analysis

All data are expressed as the mean ± standard deviation (SD). Two‐sided unpaired Student's *t* test or one‐way ANOVA were used to determine significant differences between two sets of data. *p* < 0.05 was considered statistically significant.

## 3 Results

### 3.1 PrP^C^ Expression Increases During Myotube Formation by Correlating With MyoG Expression

**FIGURE 1:**
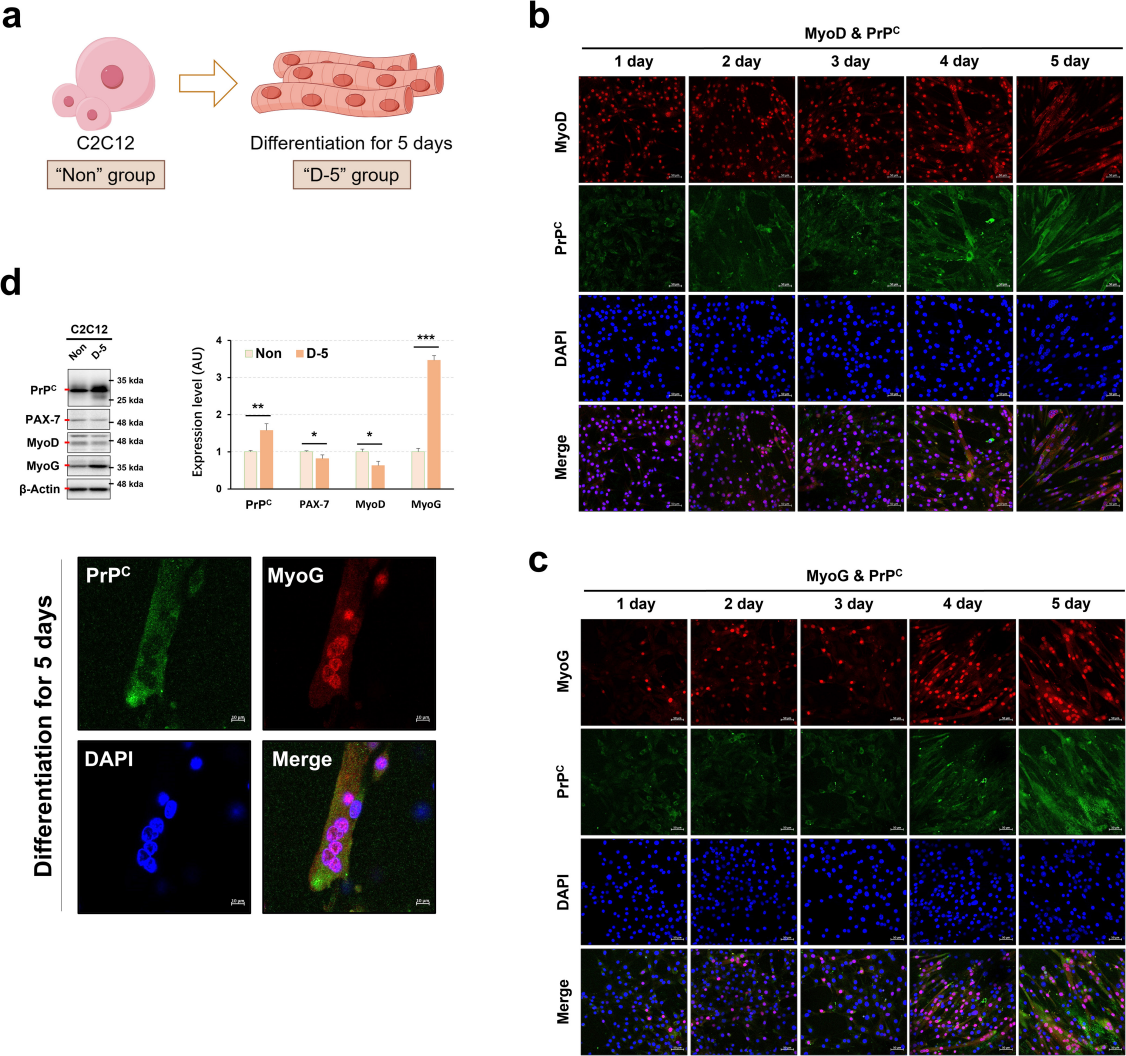
PrP^C^ expression becomes active during myoblast differentiation. (a) Schematic diagram of C2C12 cell differentiation and grouping (By Figdraw, UAYYR9d9d0). (b,c) The expression levels of MyoD/PrP^C^ (b) and MyoG/PrP^C^ (c) were visualized at the indicated times during myoblast differentiation by immunofluorescence; a representative result from three independent experiments is shown (technical replicates). (d) The expression levels of PrP^C^, Pax7, MyoD and MyoG proteins were measured at 5 days post‐incubation of C2C12 cells under differentiation medium by the analysis of western blotting (upper panel). Immunofluorescence images of PrP^C^ and MyoG protein at 5 days post‐incubation of C2C12 cells under differentiation medium (lower panel); a representative result from three independent experiments is shown (technical replicates). Data are presented as mean ± SD. Data was analysed by two‐sided unpaired Student's *t* tests (* *p* < 0.05; ** *p* < 0.01; *** *p* < 0.001; ns, not significant, *p* > 0.05).

To investigate the role of PrP^C^ in muscle homeostasis, we assessed the change of its expression during muscle formation using C2C12 cell line (Figure 1a). As shown in Figure 1b,c, the expression of PrP^C^ was more intensified during the process of myotube formation, exhibiting a close correlation with MyoG expression. This relationship was distinct from that observed with myogenic differentiation (MyoD), which displayed an opposite trend throughout myoblast differentiation. These results were more evidenced in western blotting and immunofluorescence analyses (Figure 1d). These findings indicate that PrP^C^ can be involved in skeletal muscle homeostasis by modulating myotube formation.

### 3.2 PrP^C^ Plays a Crucial Role in Maintaining Muscle Homeostasis, Particularly in Pre‐Aging Adulthood, by Limiting Reduced Muscle Growth or Mass Accumulation, and Influencing Muscle Regeneration and Function

**FIGURE 2:**
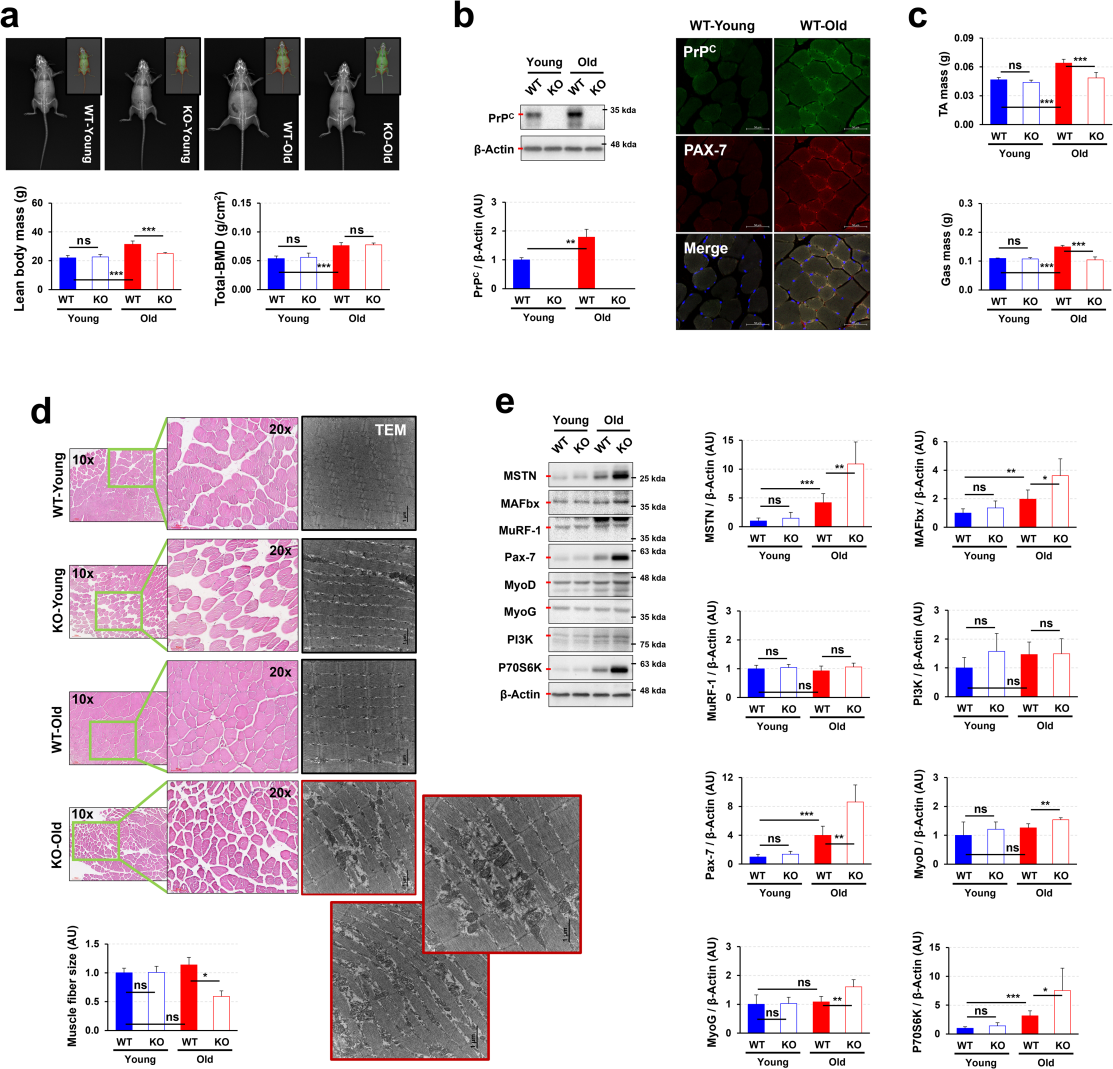
PrP^C^ deficiency accelerates skeletal reduced muscle growth or mass accumulation during ageing. (a) Lean body weight and bone mineral density were analysed by Dual‐Energy X‐ray Absorptiometry (DEXA); a representative result is shown (*n* = 6). (b) The expression level of PrP^C^ was measured in the gastrocnemius muscles of both young and old WT and KO mice by analysis of western blotting; a representative result is shown (*n* = 6). Pax‐7 and PrP^C^ expression in the tibialis anterior muscles of young and old WT mice were visualized using immunofluorescence. (c) Mass of tibialis anterior (TA) and gastrocnemius (Gas) muscles (*n* = 6). (d) Muscle fibre size in tibialis anterior muscle and muscle fibre images for extensor digitorum longus muscle were measured by H&E staining and TEM, respectively (*n* = 6). (e) The expression levels of MSTN, MAFbx, MuRF‐1, Pax‐7, MyoD, MyoG, PI3K and P70S6K were measured in the gastrocnemius muscles of WT and KO mice by analysis of western blotting; a representative data is shown (*n* = 6). Data are presented as mean ± SD. Data was analysed by one‐way ANOVA or two‐sided unpaired Student's *t* tests (* *p* < 0.05; ** *p* < 0.01; *** *p* < 0.001; ns, not significant, *p* > 0.05).

We next investigated the role of PrP^C^ in muscle homeostasis throughout the life span. For this, we facilitated *Prnp* WT and KO mice knockout with PrP^C^, depending on age (Figures S1a and S2b). Lean body mass and bone mineral density (BMD) significantly increased with age (Figure 2a). However, body weight was reduced in old KO mice compared to their corresponding WT mice, while no such reduction was observed in young KO mice. Active muscle formation is needed for compensating muscle loss in the body with old age rather than young age [37]. As shown in Figure 2b, the expression level of PrP^C^ was significantly increased in old WT mice compared to young WT mice, paralleling the expression of the satellite cell marker, Pax7. Based on this result, we can hypothesize that PrP^C^ plays an important role in maintaining muscle homeostasis in pre‐ageing adulthood, more so than in youth. This hypothesis was supported by our finding that the mass of TA and Gas muscles statistically decreased in old KO mice, but not in young KO mice, compared to corresponding WT mice (Figure 2c). Further, only old KO mice, but not young KO mice, exhibited a significantly reduced size of muscle fibre and even possessed fibre damage, as evidenced by TEM (Figure 2d).

The muscle atrophy‐related phenotype observed in older KO mice was further characterized by increased expression levels of myostatin (MSTN) and muscle atrophy F‐box (MAFbx), both of which are negative regulators of skeletal muscle mass [38] (Figure 2e). In contrast, the expression level of muscle‐specific RING finger protein 1 (MuRF1), another factor associated with atrophy, did not change in older KO mice compared to their WT counterparts (Figure 2e). It has been reported that the activation of phosphoinositide 3‐kinase (PI3K) can induce muscle hypertrophy [39]. However, the expression of PI3K did not differ in older KO mice compared to their WT counterparts (Figure 2e). In older KO mice with a skeletal muscle injury, we observed a significant upregulation of P70S6K, a protein synthesis‐regulating factor, along with increased expression of muscle differentiation‐related factors such as Pax‐7, MyoD and MyoG. Although skeletal muscle in the injured state attempts to regulate tissue regeneration in the injured region [40], this process is ineffective in older KO mice. These results in old KO mice were attended by an endurance capacity test and grip strength test. In contrast to young KO mice, old KO mice displayed a statistically significant decrease in endurance capacities such as continuous running time, vertical running distance and total work and grip strength, compared to corresponding WT mice (Figure S1b,c). These findings suggest that PrP^C^ is important for skeletal muscle homeostasis by limiting muscle atrophy, especially in pre‐ageing adulthood.

### 3.3 PrP^C^ Deficiency Disrupts Mitochondrial Homeostasis by Unbalancing Mitophagy and Mitochondrial Biogenesis, Leading to Mitochondrial Damage in Skeletal Muscle, Especially in Pre‐Aging Adulthood

**FIGURE 3:**
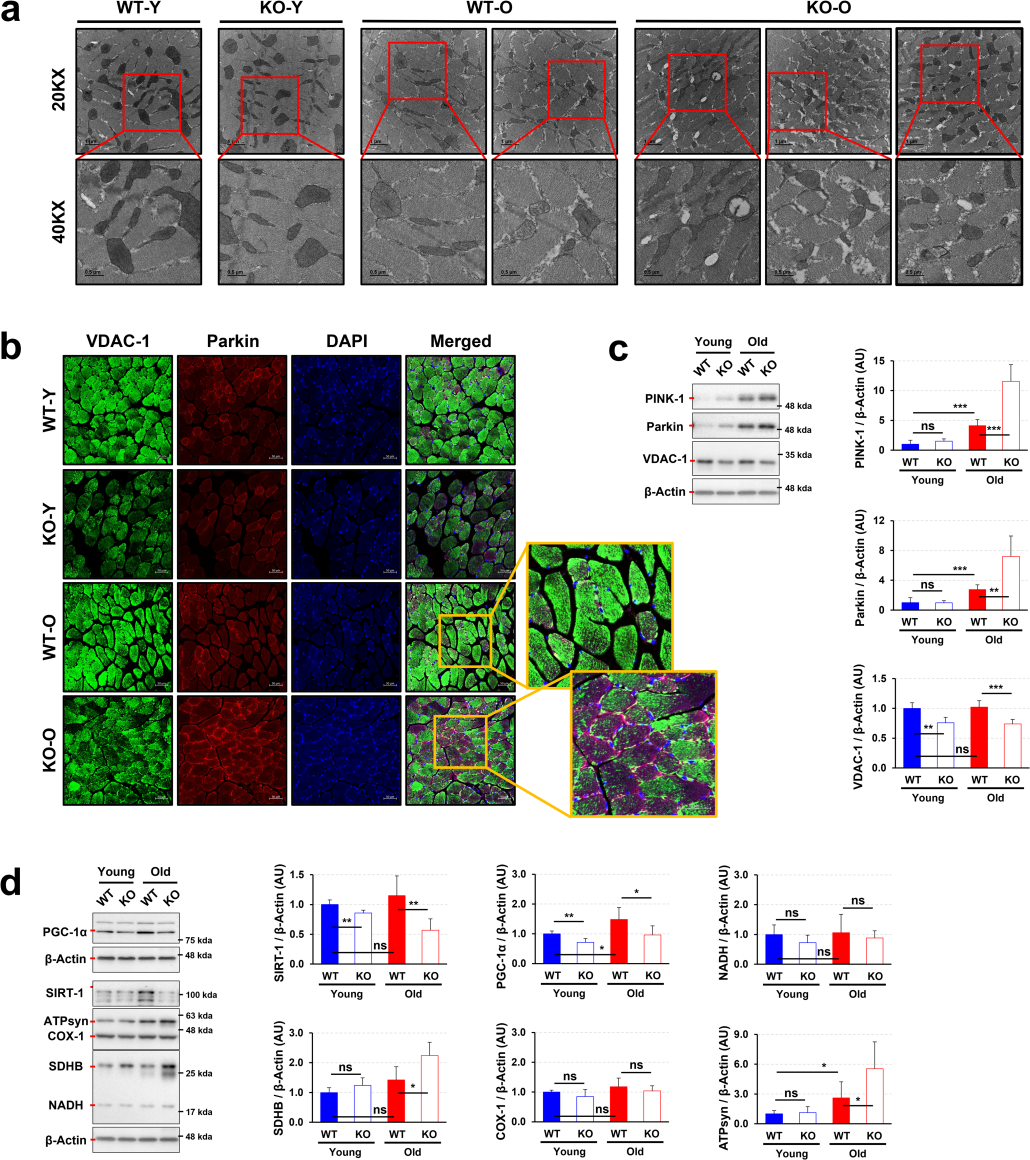
PrP^C^ deficiency disturbs the homeostasis of mitochondria by modulating mitophagy and mitochondrial biogenesis. (a) Mitochondrial images were measured in the extensor digitorum longus muscles of young and old WT and KO mice by analysis of TEM. (b) The expression levels of VDAC‐1 and Parkin were visualized in the tibialis anterior muscles of the mice using immunofluorescence; a representative result from three independent experiments is shown. (c) The expression levels of PINK‐1, Parkin and VDAC‐1 were assessed in the gastrocnemius muscles of WT and KO mice by western blotting; a representative data is shown (*n* = 6). (d) Protein expression levels of SIRT‐1, PGC‐1*α* and OXPHOS (NADH‐UO, SDHB, COX‐1 and ATPsyn) in gastrocnemius muscle were shown by analysis of western blotting; a representative data is shown (*n* = 6). Data are presented as mean ± SD. Data was analysed by one‐way ANOVA or two‐sided unpaired Student's *t* tests (* *p* < 0.05; ** *p* < 0.01; *** *p* < 0.001; ns, not significant, *p* > 0.05).

Mitochondria are the cellular modulators for maintaining skeletal muscle homeostasis^S1^. Thus, we next investigated whether the observed muscle fibre injury in old KO mice was linked to mitochondrial damage. TEM analysis revealed that while young KO mice exhibited mitochondria with a phenotype comparable to WT controls, aged KO mice displayed significant mitochondrial abnormalities. These abnormalities included disruption of the mitochondrial outer membrane and notable morphological anomalies (Figure 3a). Mitophagy, a selective autophagic process, is essential for cellular homeostasis by eliminating dysfunctional mitochondria^S2^. As expected, significantly increased levels of mitophagy‐related factors such as PTEN‐induced kinase 1 (PINK1) and Parkin were observed in the gastrocnemius muscle of old KO mice, but not of young KO mice, compared to that of corresponding WT mice (Figure 3c).

This observation is consistent with previous reports indicating that reduced levels of voltage‐dependent anion‐selective channel 1 (VDAC‐1), a key substrate of Parkin, promote enhanced mitophagy^S3^. VDAC‐1 levels were lower in KO mice, irrespective of age (Figure 3b,c), contributing directly to mitochondrial dysfunction and excessive mitophagy in ageing. Mitochondrial homeostasis requires a balance between mitophagy and mitochondrial biogenesis^S4^. Of note, the expression of mitochondrial biogenesis‐related factors such as peroxisome proliferator‐activated receptor‐gamma coactivator (PGC)‐1*α* and NAD‐dependent protein deacetylase sirtuin‐1 (SIRT‐1) was significantly decreased in young and old KO mice compared to each corresponding WT mice (Figure 3d). As a result, this imbalance between mitophagy and biogenesis in the skeletal muscle of KO mice ultimately compromised mitochondrial function, despite the continued upregulation of mitophagy‐related processes. With continued low‐level expression of PGC‐1*α*, Sirt‐1 & VDAC‐1, the oxidative phosphorylation (OXPHOS)‐associated factors ATP synthase (ATPsyn) and succinate dehydrogenase iron–sulphur subunit B (SDHB) appeared to be more active in old KO mice than in corresponding WT mice (Figure 3d). However, no significant differences were observed in the expression of COX‐1 or NADH dehydrogenase between WT and KO mice (Figure 3d). These findings suggest that PrP^C^ deficiency causes the disruption of mitochondrial homeostasis by impairing balance between mitophagy and mitochondrial biogenesis.

### 3.4 PrP^C^ Deficiency in pre‐Aging Adulthood but Not Young age Leads to Mitochondrial Damage by Activating Oxidative Stress

**FIGURE 4:**
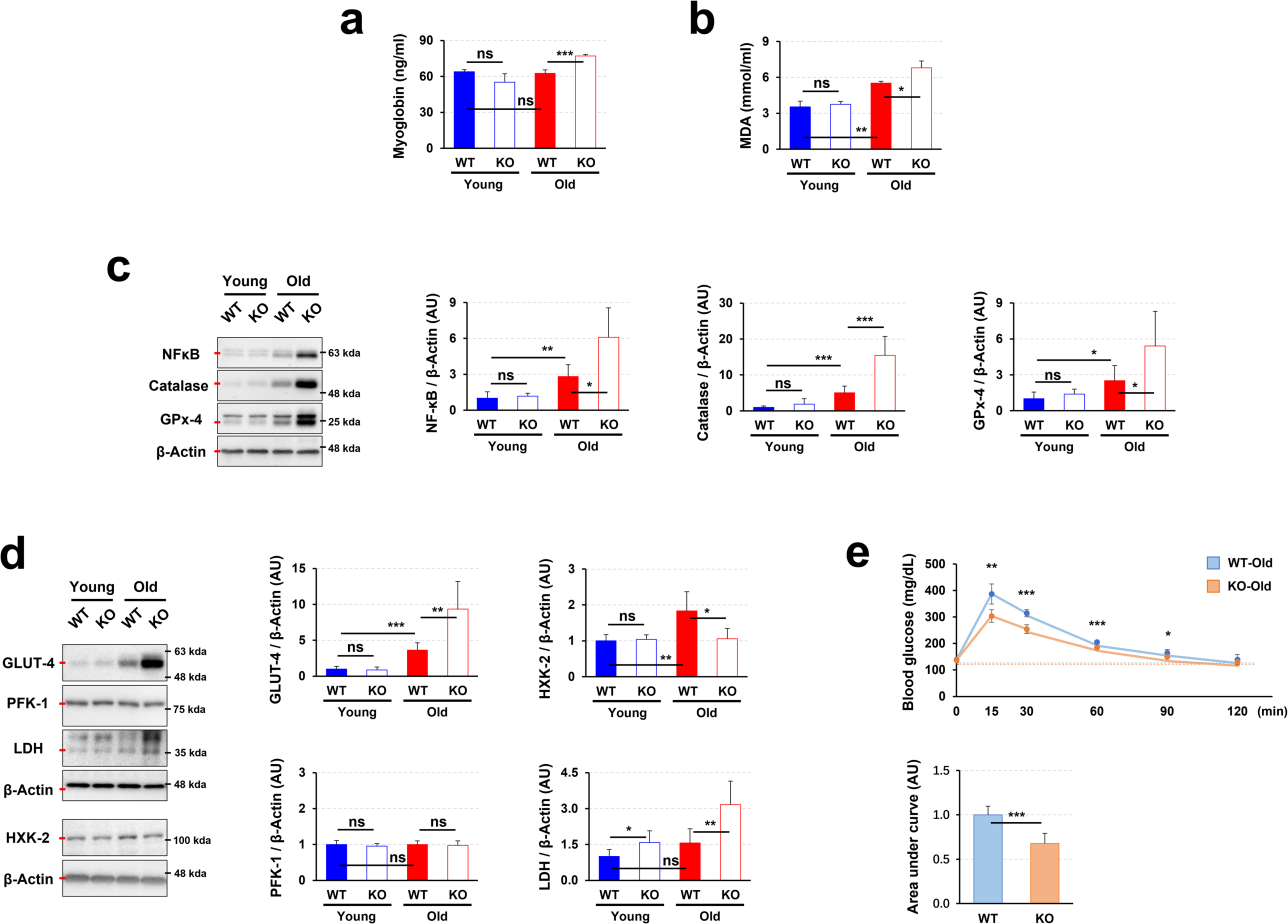
PrP^C^ deficiency enhance oxidative stress and skeletal muscle glucose metabolism. (a) Serum Myoglobin Levels were measured in the blood of the mice; a representative data is shown (*n* = 4). (b) MDA levels were measured in the gastrocnemius muscles of the mice by TBARs assay; a representative data is shown (*n* = 6). (c) The expression levels of NF‐κB, Catalase and GPx4 were measured in the gastrocnemius muscles of the mice by analysis of western blotting; a representative data is shown (*n* = 6). (d) The expression levels of GLUT‐4, HXK‐2, PFK‐1 and LDH were measured in the gastrocnemius muscles of young and old WT and KO mice by analysis of western blotting; a representative result is shown (*n* = 6). (e) Results of the oral glucose tolerance test were shown (*n* = 6). Data are presented as mean ± SD. Data was analysed by one‐way ANOVA or two‐sided unpaired Student's *t* tests (* *p* < 0.05; ** *p* < 0.01; *** *p* < 0.001; ns, not significant, *p* > 0.05).

A mitochondrion is a place for providing both the crucial sources and the main targets of reactive oxygen species (ROS)^S5^. In stressful conditions, unlike homeostatic conditions, mitochondrial ROS evokes oxidative damage and leads to mitochondrial dysfunction that produces even more ROS^S6^. To explore the relationship between PrP^C^ deficiency‐induced mitochondrial damage and ROS accumulation in pre‐ageing adulthood, we measured serum myoglobin level, which is used for determining oxidative stress‐induced muscle damage^S7^. A significantly higher level of myoglobin was detected in the blood of old KO mice compared to their WT counterparts, while young KO mice did not exhibit this increase (Figure 4a). Malondialdehyde (MDA), a well‐established marker of lipid peroxidation marker and oxidative stress^S8^, is increased with age and is more enhanced in old KO mice than corresponding WT mice (Figure 4b). NF‐κB plays a protective role in the modulation of ROS accumulation under the conditions of oxidative stress^S9^. It is well known that ageing is accompanied by enhanced oxidative stress^S10^. As expected, old WT mice had a significantly increased expression level of NF‐κB, compared to young WT mice. Its expression levels became stronger in old KO mice (Figure 4c). In a similar pattern, the antioxidant enzymes catalase and glutathione peroxidase‐4 (GPx‐4), which catalyse the breakdown of hydrogen peroxide into water, also displayed age‐dependent increases. These enzymes were significantly more elevated in aged KO mice compared to their WT counterparts (Figure 4c). These findings suggest that PrP^C^ deficiency activates oxidative stress in skeletal muscle of pre‐ageing adulthood.

### 3.5 PrP^C^ Deficiency in pre‐Aging Adulthood Leads to a Compensatory Increase in Skeletal Muscle Glucose Metabolism

Mitochondrial damage and abnormality demand skeletal muscle glucose metabolism^S11^. As expected, in aged KO mice, key markers of muscle glucose homeostasis, including Glucose Transporter Type 4 (GLUT4) and Lactate Dehydrogenase (LDH), were significantly upregulated compared to the corresponding WT mice (Figure 4d, upper panel). Interestingly, despite increased glucose uptake, the conversion of glucose to glucose‐6‐phosphate was restricted by reduced expression of Hexokinase‐2 (HXK‐2), resulting in a lack of upregulation of glycolytic enzymes such as Phosphofructokinase‐1 (PFK‐1), which did not respond to the heightened metabolic demand (Figure 4d, lower panel). This limitation in glycolytic flux appeared to be partially offset by the increased expression of LDH, suggesting a compensatory shift towards lactate metabolism to meet the sustained energy requirements (Figure 4d). Moreover, the oral glucose tolerance zero test showed that a significantly impaired glucose tolerance in aged KO mice compared to their WT counterparts (Figure 4e), providing direct support for the idea of a compensatory increase in glucose metabolism to meet the increased energy demand in old KO group.

### 3.6 PrP^C^ Deficiency Leads to Reduced Muscle Growth or Mass Accumulation by Disrupting Both ER and Mitochondrial Function During the Aging Process, Particularly in Late Middle age

**FIGURE 5:**
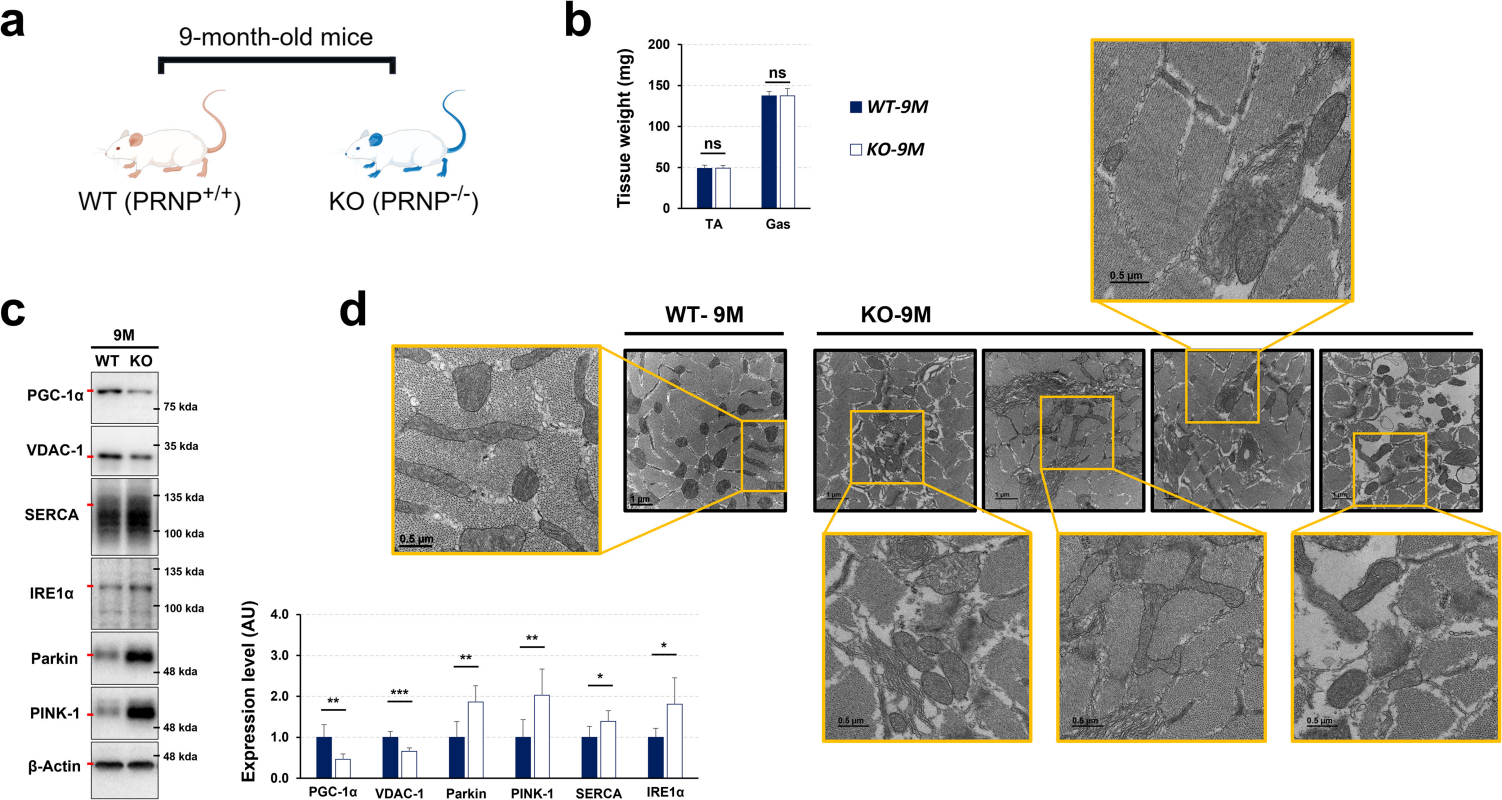
PrP^C^ deficiency causes mitochondrial damage and ER stress. (a) Schematic diagram of mouse grouping (By Figdraw, UAYYR9d9d0). (b) Weight of TA and Gas muscles in 9 months mice (*n* = 6). (c) Protein expression levels of PGC‐1*α*, VDAC‐1, Parkin, PINK‐1, SERCA and IRE1*α* in gastrocnemius muscles by western blotting in 9 months mice; a representative data is shown (*n* = 6). (d) Images for extensor digitorum longus muscle by TEM in 9 month mice. Data are presented as mean ± SD. Data was analysed by two‐sided unpaired Student's *t* tests (* *p* < 0.05; ** *p* < 0.01; *** *p* < 0.001; ns, not significant, *p* > 0.05).

Under ER stress, mitochondrial dysfunction is accelerated^S12^. Thus, we next questioned how PrP^C^ deficiency‐induced reduced muscle growth or mass accumulation with mitochondrial dysfunction in late middle‐age, correlates with ER stress. To answer this question, we studied KO mice of 9 months old (Figure 5a), which did not change in tibialis anterior (TA), and gastrocnemius (Gas) muscle mass compared to the corresponding WT mice (Figure 5b). Similar to 15‐month‐old KO mice, 9‐month‐old KO mice exhibited higher levels of mitophagy‐related factors such as PINK‐1 and Parkin, and lower expression levels of mitochondrial function‐related factors such as PGC‐1*α* and VDAC‐1 than corresponding WT mice (Figure 5c). Different from WT mice, the KO mice exhibited an ER stress‐related phenotype with increased number, as evidenced by TEM analysis (Figure 5d). Expression levels of sarcoplasmic/endoplasmic reticulum CA2^+^‐ATPase (SERCA) and inositol‐requiring transmembrane kinase/endonuclease 1 alpha (IRE1 alpha), indicators of ER stress, provide a more specific indication of ER status. SERCA expression was significantly elevated in both young and 9‐month‐old KO mice compared to WT mice but declined in older KO mice (Figures 5c and S2), suggesting an early compensatory response to disrupted dynamic calcium signalling. In contrast, IRE1 alpha levels were not different in young mice but were significantly higher in 9‐ and 15‐month‐old KO mice than in WT mice (Figures 5c and S2), indicating progressive ER stress with ageing. Collectively, these findings illustrate that PrP^C^ deficiency causes reduced muscle growth or mass accumulation by disturbing ER and mitochondria function.

### 3.7 PrP^C^ Deficiency Accelerates Senescence of Satellite Cells With the Impairment of Their Regenerative Potential

**FIGURE 6:**
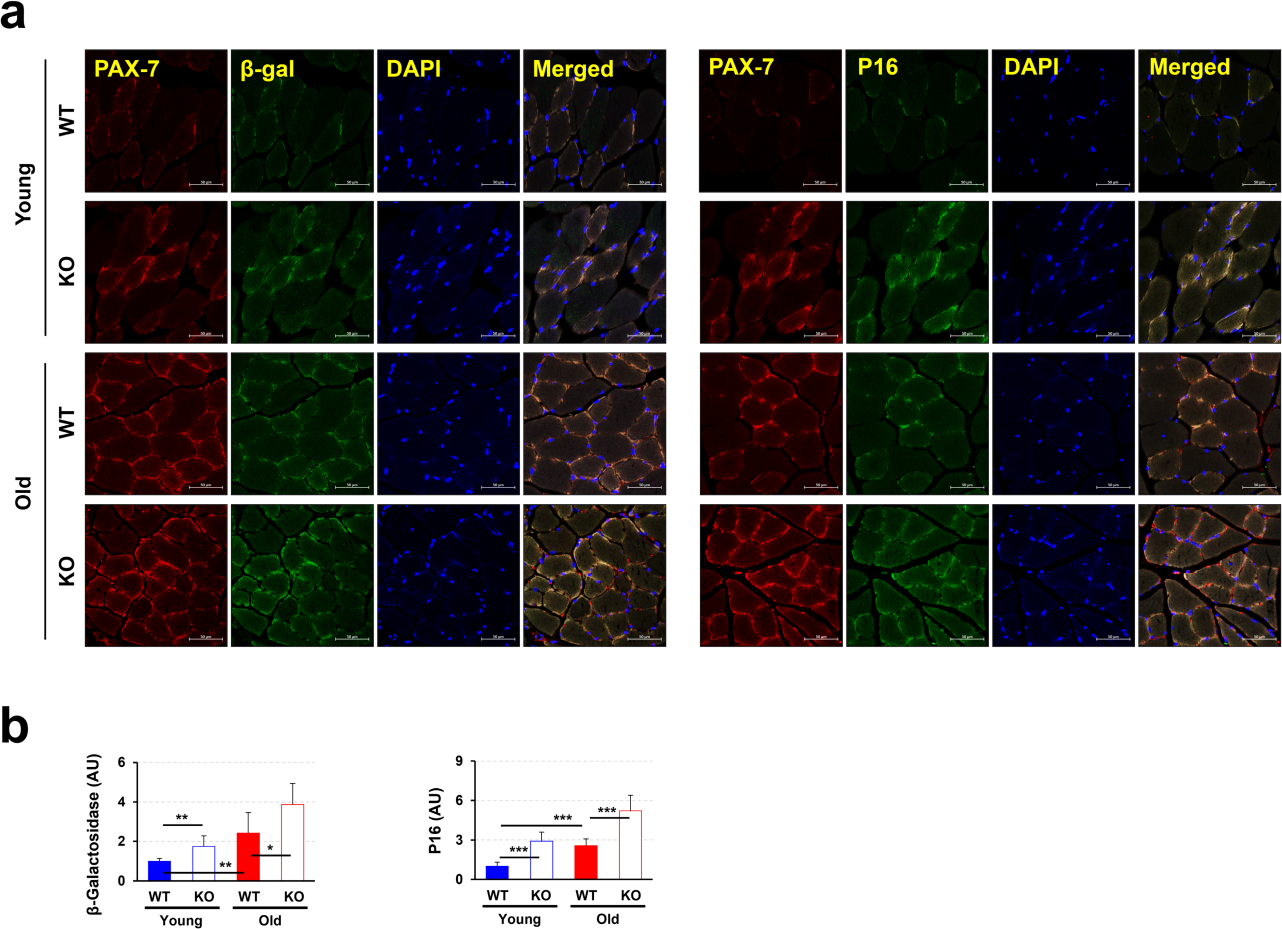
PrP^C^ deficiency causes SC senescence. (a) The expression levels of Pax‐7/*β*‐galactosidase and Pax‐7/p16 were measured in the tibialis anterior muscles of young and old WT and KO mice by immunofluorescence; a representative data is shown (*n* = 3). (b) The expression levels of *β*‐galactosidase and p16 were evaluated in the tibialis anterior by immunofluorescence (*n* = 3). Data are presented as mean ± SD. Data was analysed by one‐way ANOVA or two‐sided unpaired Student's *t* tests (* *p* < 0.05; ** *p* < 0.01; *** *p* < 0.001; ns, not significant, *p* > 0.05).

Skeletal muscle growth and regeneration are modulated by SCs that are located beneath the basal lamina of muscle fibres and have a remarkable ability to self‐renew, expand and proliferate as myoblast and myogenic differentiation to fuse and restore damaged muscle^S13^. In line with this, we questioned how the status of SCs is maintained in the condition of reduced muscle growth or mass accumulation that appeared in old KO mice and why the cells did not show their regenerative potential to recover the damaged muscle. To resolve this, we first estimated the frequency of SCs expressing Pax7^S14^. During ageing, the number of stem cells increases and become more prone to senescent^S15^. As expected, Pax7‐expressing SCs increased in aged mice, with substantial overlap with PrP^C^‐expressing cells (Figures 6a,b and 2b), indicating that the loss of skeletal muscle during ageing is accompanied by the enhancement of SC expansion to restore and the existence of a role of PrP^C^ in SCs for muscle homeostasis. Similarly, PrP^C^ expression was significantly elevated in old WT mice compared to young WT mice, while aged KO mice exhibited a markedly higher number of Pax7‐expressing SCs compared to age‐matched WT mice (Figures 6a,b). During ageing and expansion, adult stem cells undergo cellular senescence, and the levels of senescence‐associated (SA)‐*β*‐galactosidase (SA‐*β*‐gal) activity and p16 become more active in the progression of stem cell senescence^S16^. As expected, the increased numbers of SA‐*β*‐gal‐ and p16‐expressing cells were found in old WT mice compared to young WT mice (Figures 6a,b). Of note, Pax7‐expressing SCs derived from both young and old KO mice possessed a significantly increased number in SA‐*β*‐gal‐ and p16‐expressing cells, compared to those derived from each corresponding WT mice (Figure 6a). These findings suggest that PrP^C^ deficiency accelerates the senescence of SCs.

**FIGURE 7:**
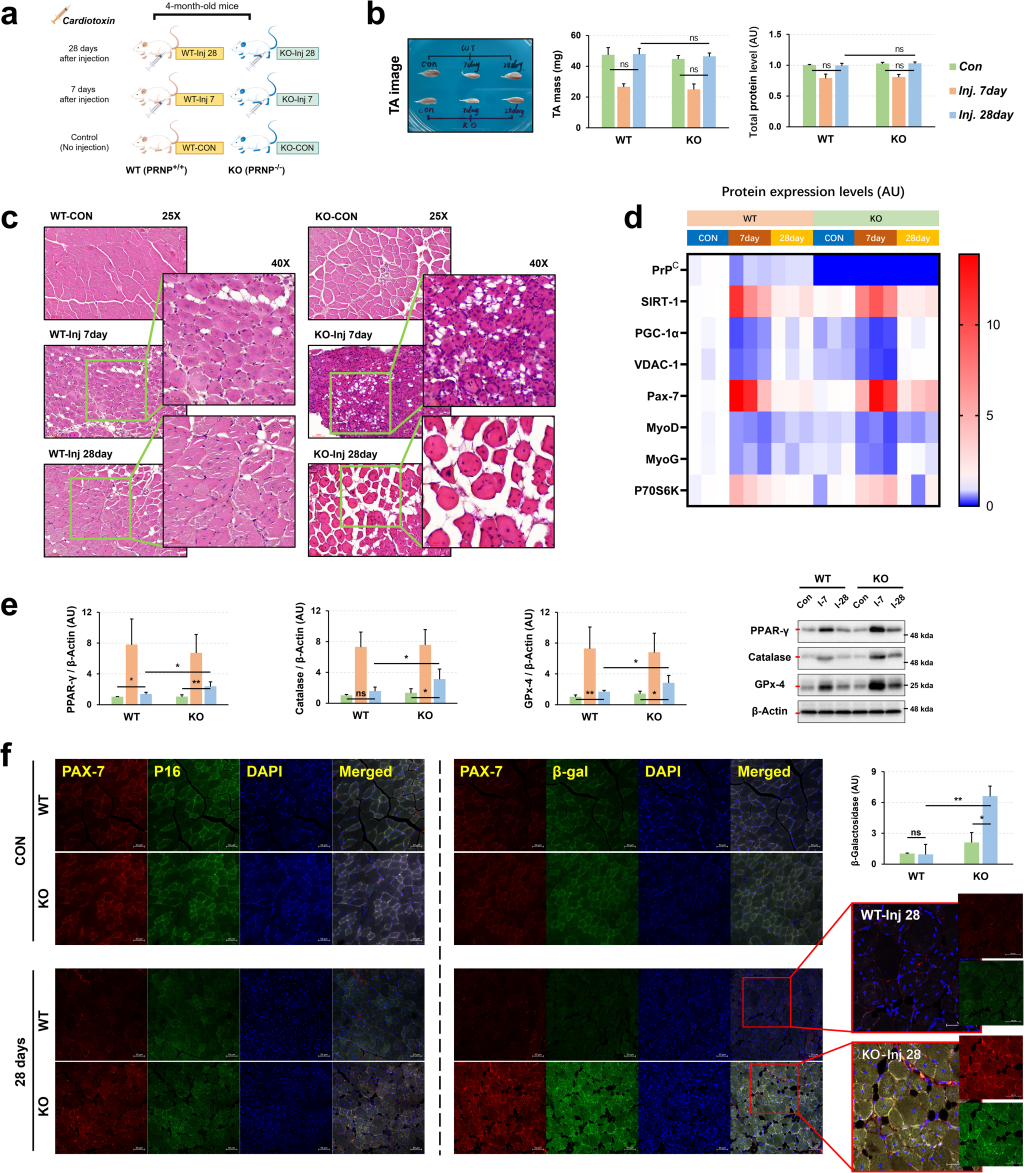
PrP^C^ deficiency causes the defect in muscle regenerative capacity. (a) Schematic diagram of mouse treatment and grouping (By Figdraw, UAYYR9d9d0). (b) Tibialis anterior muscle contrast image, tibialis anterior muscle weight, and protein levels were shown at 7 and 28 days after cardiotoxin injection (*n* = 5). (c) Muscle regeneration capacity was measured in the tibialis anterior muscles of WT and KO mice at the indicated times after cardiotoxin injection by H&E staining; a representative data is shown (*n* = 3). (d) The expression levels of PrP^C^, SIRT‐1, PGC‐1*α*, VDAC‐1, Pax‐7, MyoD and MyoG were evaluated in the tibialis anterior of the mice at the indicated times after cardiotoxin injection by western blotting (*n* = 3, a representative result image is shown in Figure S3). (e) The expression levels of PPAR*γ*, Catalase and GPx‐4 were evaluated in the tibialis anterior of the mice at the indicated times after cardiotoxin injection by western blotting: A representative data is shown (*n* = 5). (*f*) The expression levels of Pax‐7/*β*‐galactosidase and Pax‐7/p16 were evaluated in the tibialis anterior of the mice at 28 days after cardiotoxin injection by immunofluorescence; a representative data is shown (*n* = 3). Data are presented as mean ± SD. Data was analysed by one‐way ANOVA or two‐sided unpaired Student's *t* tests (* *p* < 0.05; ** *p* < 0.01; *** *p* < 0.001; ns, not significant, *p* > 0.05).

Senescent stem cells possess the defective function to regenerate damaged tissues^S17^. Thus, we next investigated the functional potential of PrP^C^ deficiency‐induced senescence of SCs in the muscle tissue injured by cardiotoxin intramuscular injection and then performed muscle recover‐associated experiments after 7 and 28 days post‐injection (Figure 7a). After 7 days, tibialis anterior (TA) muscle mass and total protein levels were significantly reduced, but both returned to normal levels by 28 days, with WT and KO mice demonstrating comparable recovery capacity (Figure 7b). Of note, KO mice formed a remarkably higher adipogenesis in cardiotoxin‐injected muscles than WT mice, as occurred in senescent mesenchymal stem cells‐mediated microenvironment^S18^ (Figure 7c). There is no significant difference in the regulation of mitochondrial biogenesis and mechanisms related to satellite cell differentiation during the damage and recovery process after injection of cardiotoxin in WT and KO mice (Figure 7d). Interestingly, although naturally ageing KO mice consistently showed significantly reduced expression of mitochondrial biogenesis markers PGC‐1*α* and VDAC‐1 in skeletal muscle (Figures 3c,d and 5c), these markers were regulated at similar levels in both KO and WT mice during muscle repair after cardiotoxin‐induced injury (Figure 7d). Nevertheless, the cardiotoxin‐injected muscles of KO mice showed an evident increase in the number of Pax7‐expressing SCs and the expression level of Pax7 until 28 days post‐infection compared to control muscle of KO mice, while the cardiotoxin‐injected muscles of WT mice exhibited a comparable level both at 28 days post‐injection, compared to control muscles of WT mice (Figure 7d,f). The cardiotoxin injection‐induced severe adipogenesis in the muscles of KO mice was correlated with the significantly increased expression of PPAR*γ*, a master regular of adipogenesis^S19^ (Figure 7e). In a similar trend, the expression levels of catalase and GPx‐4 in the cardiotoxin‐injected muscles of KO mice were also higher than in those of WT mice at 28 days post‐injection (Figure 7e). While there was no significant difference in Pax7 expression in stem cells compared to p16 expression in stem cells from WT or KO mouse muscles after 28 days of recovery (Figure 7f), Pax7‐expressing SCs derived from the cardiotoxin‐injected muscles of KO mice but not of WT mice showed an enhanced SA‐*β*‐gal activity compared to those derived from the control muscles of KO mice (Figure 7f). These findings illustrate that PrP^C^ deficiency delays or impairs complete recovery from muscle injury by accelerating SC senescence‐caused adipogenesis.

## 4 Discussion

Muscle homeostasis refers to the dynamic balance maintained within muscle tissue to ensure proper function, structure, and repair. This involves various physiological processes, including muscle protein turnover^S20^, regulation by satellite cells^S21^, signal transduction pathways and nutrient availability^S22^, to maintain muscle size, strength and performance. Notably, as ageing progresses, there is a natural decline in muscle mass and strength, a phenomenon known as sarcopenia. Diseases such as muscular dystrophy, cachexia and chronic illnesses can also disrupt muscle homeostasis, leading to reduced muscle growth or mass accumulation and weakness^S23^.

The involvement of PrP^C^ in skeletal muscle homeostasis is underscored by its expression in muscle tissue, its role in muscle formation, its participation in key signal transduction pathways, and its protective effects on muscle cells, supported by findings from experimental models. By modulating myotube formation, promoting cell survival, and limiting muscle atrophy, PrP^C^ contributes to the dynamic balance required for proper muscle function, structure, and repair. This evidence collectively highlights its significant role in muscle biology and its potential as a therapeutic target for muscle‐related diseases, including in pre‐ageing adulthood when muscles are more prone to degeneration. Our research results, as shown in Figure 1, and Figure S1, further suggest PrP^C^'s importance in skeletal muscle homeostasis, particularly in preventing muscle atrophy, especially in the elderly.

The role of PrP^C^ in maintaining mitochondrial homeostasis is crucial, as evidenced by its presence in mitochondria^S24^, its influence on mitophagy (Figure 3b,c) and mitochondrial biogenesis (Figure 3d), its antioxidant properties (Figures 4b,c), and findings from *Prnp* KO model. By regulating the balance of mitophagy and mitochondrial biogenesis, PrP^C^ ensures proper mitochondrial function and quality. PrP^C^ deficiency disrupts this balance, leading to mitochondrial dysfunction and cellular energy deficits. Our research results suggest that PrP^C^ deficiency causes mitochondrial homeostasis disruption through imbalances in mitophagy and mitochondrial biogenesis, emphasizing its potential as a therapeutic target for preserving mitochondrial function (Figure 3a–d). Additionally, PrP^C^'s role in regulating oxidative stress in skeletal muscle, particularly in pre‐ageing adulthood, is supported by its expression in muscle tissue, antioxidant properties, increased oxidative stress in PrP^C^‐deficient models, and exacerbation of age‐related oxidative damage (Figure 4a–c). By maintaining antioxidant defences and mitochondrial function, PrP^C^ protects skeletal muscle from oxidative stress, highlighting its critical role in mitigating oxidative stress, especially in ageing. Furthermore, PrP^C^ deficiency in old mice triggers compensatory increases in glucose metabolism indicators such as GLUT4 and LDH, while decreased expression of HXK‐2 limits glucose utilization efficiency, ultimately impacting oral glucose tolerance (Figure 4d,e). These findings underscore the intricate relationship between mitochondrial function, glucose metabolism indicators, and glucose tolerance in PrP^C^‐deficient mice, providing insights into PrP^C^'s role in maintaining metabolic homeostasis. Follow‐up studies using time‐course experiments measuring mitochondrial function and glucose metabolism at different stages of ageing would help clarify whether these effects are primary or secondary consequences of PrP^C^ deficiency. Lastly, PrP^C^ deficiency disrupts both ER and mitochondrial function in muscle cells, leading to reduced muscle growth or mass accumulation (Figures 5a–d and S2). This highlights PrP^C^'s importance in maintaining the functional integrity of these organelles and its potential as a therapeutic target for preventing muscle atrophy.

PrP^C^ deficiency accelerates SC senescence, impairing their regenerative capacity and contributing to age‐related muscle decline (Figures 6 and 7). Understanding how PrP^C^ regulates SC function and senescence may offer insights into therapeutic strategies for preventing or reversing muscle ageing and degeneration. Additionally, PrP^C^ deficiency limits the complete recovery of muscle damage by promoting adipogenesis through accelerated SC senescence, leading to delayed recovery or a failure of muscle to completely recover (Figures 7c,e,f). Understanding PrP^C^'s role in satellite cell function and senescence reveals potential therapeutic targets for enhancing muscle regeneration and injury recovery. The increase in ageing SCs in *Prnp* KO mice raises similar questions. While PrP^C^ is implicated in satellite cell function, it remains unclear whether PrP^C^ loss directly causes satellite cell ageing or if this is a downstream effect of general muscle degeneration in the absence of PrP^C^. A more definitive approach would require isolating satellite cells from young and old KO mice and conducting further studies in vitro to assess their regenerative capacity and ageing markers to confirm whether PrP^C^ directly regulates cell fate.

Interestingly, stem cell senescence is often caused by an increase in mitochondrial ROS^S25^, leading to a significant increase in *β*‐galactosidase expression, which further accelerates the ageing of the cell cycle (increased expression of p16). In this study, the increased expression levels of *β*‐galactosidase and p16 (Figure 6) associated with skeletal muscle stem cell senescence coincided with low levels of mitochondrial biogenesis (Figure 3d) and abnormal calcium signalling (Figure S2). This correlation suggests that mitochondria may be a key factor in the systemic homeostasis of skeletal muscle in PrP^C^‐deficient mice. Naturally ageing *Prnp* KO mice always have significantly lower expression of the mitochondrial biogenesis regulator PGC‐1*α* (Figure 3d) and the mitochondrial membrane ion channel protein VDAC‐1 (Figure 3b,c) than their WT mice. This may be the primary cause of increased mitochondrial autophagy (Figure 3b,c), ER stress (Figure S2), and the senescence of SCs (Figure 6). The significantly low expression of the mitochondrial membrane ion channel protein VDAC‐1 (Figure 3b,c) was accompanied by a significant increase in the expression of the ER calcium signalling regulatory factor SERCA (Figure S2) in young to middle‐aged *Prnp* KO mice. The compensatory changes in the above factors explain the abnormalities in mitochondrial function and calcium signalling mechanisms in the skeletal muscle of PrP^C^‐deficient mice. As age increases, abnormal mitochondrial function and low mitochondrial biogenesis (Figure 3) contribute to the deterioration of skeletal muscle homeostasis. Starting from 9 months of age, a significant increase in the mitochondrial autophagy factor (Figures 3c and 5c) also led to more severe ER stress (increased expression of IRE‐1*α*) (Figures 5c and S2). However, the increase in mitochondrial autophagy, a protective mechanism that is supposed to remove damaged mitochondria, actually increases metabolic stress under low biogenesis conditions. This further weakens skeletal muscle homeostasis.

Although this study offers important insights into the role of PrP^C^ in skeletal muscle homeostasis, it primarily relies on correlative data. Future studies should focus on establishing clearer causal relationships by restoring PrP^C^ expressions in specific cell types like satellite cells or muscle fibres in *Prnp* KO mice. Such studies will be critical in determining whether restoring PrP^C^ expression can effectively reinstate muscle homeostasis and prevent muscle atrophy.

Our findings first demonstrate that PrP^C^ is pivotal for maintaining skeletal muscle homeostasis by modulating oxidative stress, mitochondrial function and ER stress during ageing. PrP^C^ expressed in SCs demonstrates the ability to limit their senescent process, thereby facilitating a well‐integrated recovery during regeneration after muscle injury. Further, we prove the importance of PrP^C^ in regulating exercise capacity and skeletal muscle glucose metabolism. Further study on PrP^C^'s role in exercise capacity and glucose metabolism is needed for health maintenance during ageing.

## Conflicts of Interest

The authors declare no conflicts of interest.

## Supporting information


**Figure S1.** PrP^C^ deficiency impairs exercise capacity
**Figure S2.** PrP^C^ deficiency disturbs ER.
**Figure S3.** The representative images of western blotting for Figure 7d.
