# Supplementary material for: PrPC Glycoprotein Is Indispensable for Maintenance of Skeletal Muscle Homeostasis During Aging
Source: J Cachexia Sarcopenia Muscle. 2025 Jan 28;16(1):e13706. doi: 10.1002/jcsm.13706 (PMC11773342; doi:10.1002/jcsm.13706)
Supplement: Supplementary file 1 — Figure S1. PrPC deficiency impairs exercise capacity Figure S2. PrPC deficiency disturbs ER. Figure S3. The representative images of western blotting for Figure 7d. [file JCSM-16-e13706-s001.docx]

**PrP^C^ glycoprotein is indispensable for maintenance of skeletal muscle homeostasis during aging**

Wenduo Liu^1#^, Thi Thu Trang Kieu^2#^, Zilin Wang^1^, Hyun-Jaung Sim^2,3^, Seohyeong Lee^4^, Jeong-Chae Lee^2,3^, Yoonjung Park^5^, Sang Hyun Kim^1*^, Sung-Ho Kook ^2*^

^1^Department of Sports Science, College of Natural Science, Jeonbuk National University, Jeonju 54896, Republic of Korea;

^2^Department of Bioactive Material Sciences, Research Center of Bioactive Materials, Jeonbuk National University, Jeonju 54896, Republic of Korea;

^3^Cluster for Craniofacial Development and Regeneration Research, Institute of Oral Biosciences and School of Dentistry, Jeonbuk National University, Jeonju 54896, Republic of Korea;

^4^ Department of Nutritional Sciences, University of California Berkeley, Berkeley, California, 94720, USA.

^5^Laboratory of Integrated Physiology, Department of Health & Human Performance, University of Houston, Texas, 77204, USA.

^*^Address correspondence to: Sang Hyun Kim, Department of Sports Science, College of Natural Science, Jeonbuk National University, Jeonju 54896, Republic of Korea, Phone: ++82-63-270-2853, Fax: +82-63-270-4234, E-mail: sh5275@jbnu.ac.kr

Sung-Ho Kook, Department of Bioactive Material Sciences, Jeonbuk National University, Jeonju 54896, Republic of Korea, Phone: +82-63-270-3327, Fax: +82-63-270-4312, E-mail: kooksh@jbnu.ac.kr

^#^ These authors contributed equally to this work


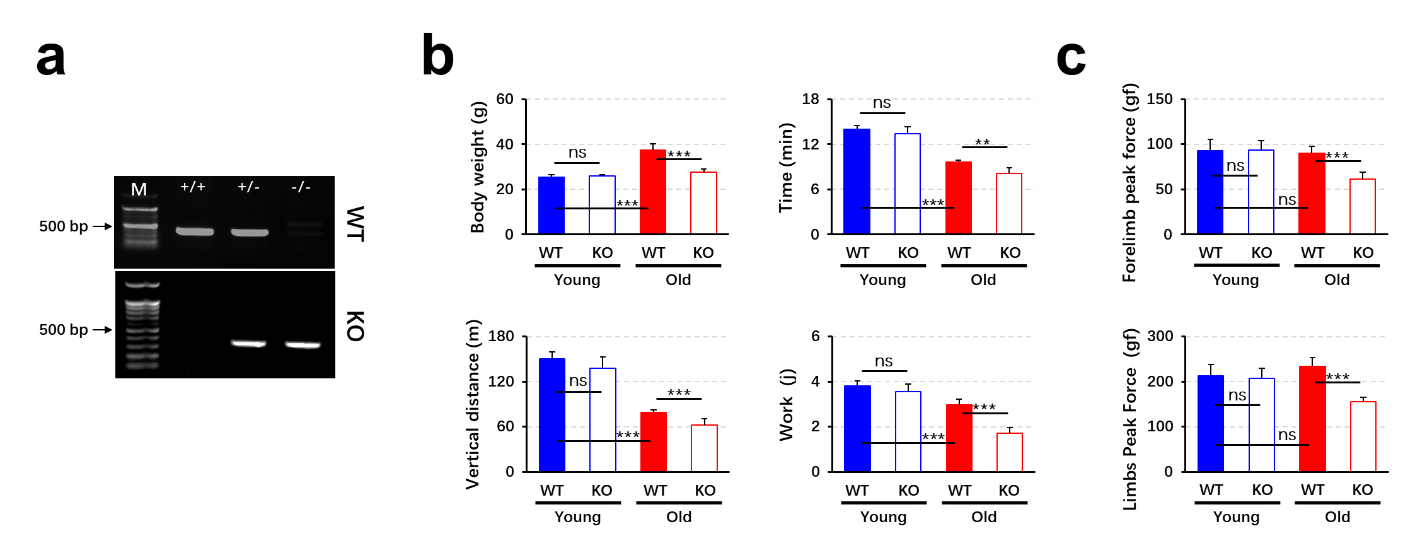


**Supplementary Fig. 1. PrP^C^ deficiency impairs exercise capacity. a** PCR genotyping for identification of *Prnp* WT and KO mice. **b** Body weight, continuous running time (time), vertical running distance (vertical distance), and total work (work) were measured in young and old WT and KO mice during the endurance capacity test on the treadmill (n = 6). **c** Grip strength test was measured in the forelimb and limb of the mice (n = 6). Data are presented as mean ± SD. Data was analyzed by one-way ANOVA or two-sided unpaired Student’s t-tests (** p＜0.01; *** p＜0.001; ns, not significant, p > 0.05).


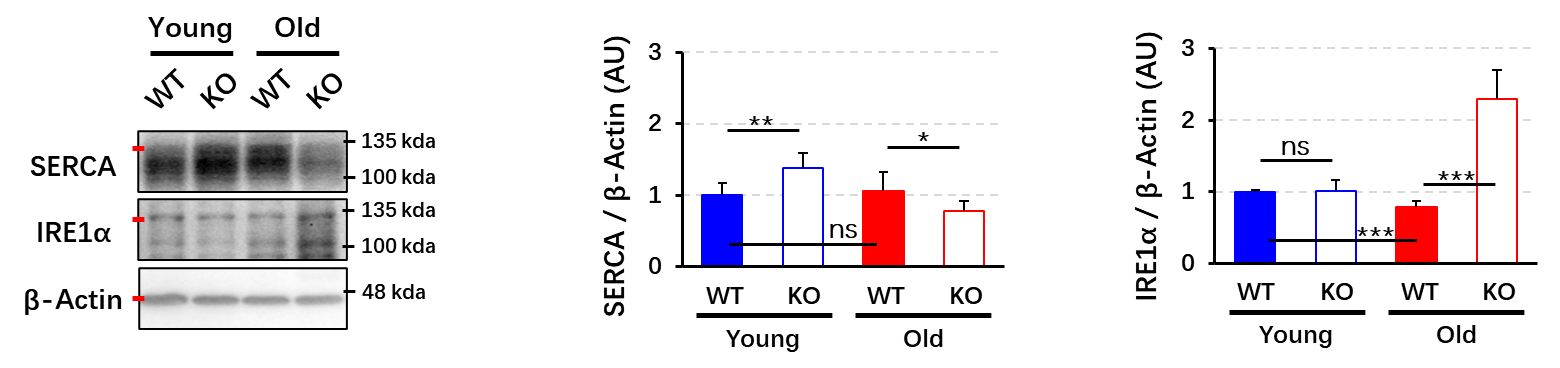


**Supplementary Fig. 2. PrP^C^ deficiency disturbs ER.** The expression levels of SERCA and IRE1α were evaluated in the gastrocnemius muscles of the mice by analysis of western blotting; a representative data is shown (n = 6). Data are presented as mean ± SD. Data was analyzed by one-way ANOVA or two-sided unpaired Student’s t-tests (* p＜0.05; ** p＜0.01; *** p＜0.001; ns, not significant, p > 0.05).

**
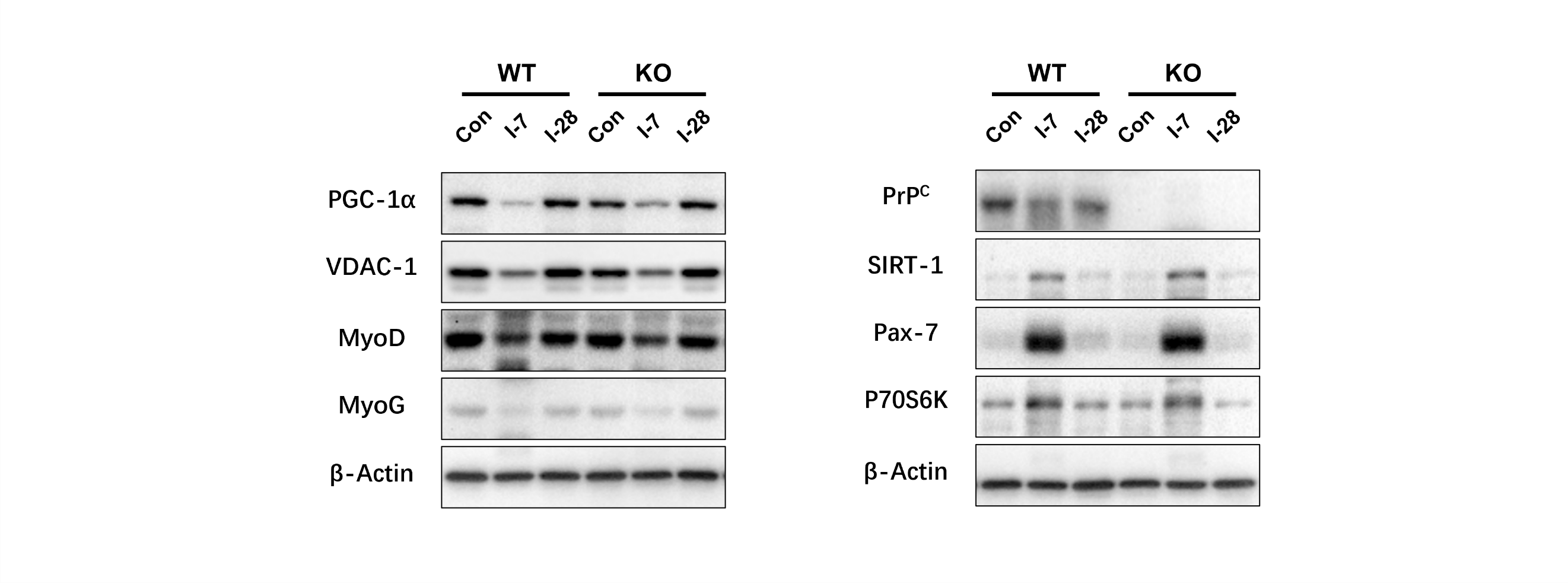
Supplementary Fig. 3. The representative images of western blotting for Fig. 7d.**

**Supplementary References**

1. Pang BPS, Chan WS, Chan CB. Mitochondria Homeostasis and Oxidant/Antioxidant Balance in Skeletal Muscle-Do Myokines Play a Role?. *Antioxidants (Basel)* 2021;**10**:179.
2. Gustafsson ÅB, Dorn GW. Evolving and Expanding the Roles of Mitophagy as a Homeostatic and Pathogenic Process. *Physiol Rev* 2019;**99**:853-892.
3. Li J, Lu J, Mi Y, Shi Z, Chen C, Riley J, et al. Voltage-dependent anion channels (VDACs) promote mitophagy to protect neuron from death in an early brain injury following a subarachnoid hemorrhage in rats. *Brain Res* 2014;**1573**:74-83.
4. Palikaras K, Tavernarakis N. Mitochondrial homeostasis: the interplay between mitophagy and mitochondrial biogenesis. *Exp Gerontol* 2014;**56**:182-188.
5. McArdle F, Pattwell DM, Vasilaki A, McArdle A, Jackson MJ. Intracellular generation of reactive oxygen species by contracting skeletal muscle cells. *Free Radic Biol Med* 2005;**39**:651-657.
6. Slimen IB, Najar T, Ghram A, Dabbebi H, Ben Mrad M, Abdrabbah M. Reactive oxygen species, heat stress and oxidative-induced mitochondrial damage. A review. *Int J Hyperthermia* 2014;**30**:513-523.
7. Brancaccio P, Lippi G, Maffulli N. Biochemical markers of muscular damage. *Clin Chem Lab Med* 2010;**48**:757-767.
8. Singh Z, Karthigesu IP, Singh P, Rupinder KAUR. Use of malondialdehyde as a biomarker for assessing oxidative stress in different disease pathologies: a review. *Iran J Public Health* 2014;**43**:7-16.
9. Lingappan K. NF-κB in Oxidative Stress. *Curr Opin Toxicol* 2018;**7**:81-86.
10. Mendoza-Núñez VM, Ruiz-Ramos M, Sánchez-Rodríguez MA, Retana-Ugalde R, Muñoz-Sánchez JL. Aging-related oxidative stress in healthy humans. *Tohoku J Exp Med* 2007;**213**:261-268.
11. Morrow RM, Picard M, Derbeneva O, Leipzig J, McManus MJ, Gouspillou G, et al. Mitochondrial energy deficiency leads to hyperproliferation of skeletal muscle mitochondria and enhanced insulin sensitivity. *Proc Natl Acad Sci* 2017;**114**:2705-2710.
12. Chen Q, Samidurai A, Thompson J, Hu Y, Das A, Willard B, et al. Endoplasmic reticulum stress-mediated mitochondrial dysfunction in aged hearts. *Biochim Biophys Acta Mol Basis Dis* 2020;**1866**:165899.
13. Ten Broek RW, Grefte S, Von den Hoff JW. Regulatory factors and cell populations involved in skeletal muscle regeneration. *J Cell Physiol* 2010;**224**:7-16.
14. Von Maltzahn J, Jones AE, Parks RJ, Rudnicki MA. Pax7 is critical for the normal function of satellite cells in adult skeletal muscle. *Proc Natl Acad Sci* 2013;**110**:16474-16479.
15. Goodell MA, Rando TA. Stem cells and healthy aging. *Science* 2015;**350**:1199-1204.
16. Lee BY, Han JA, Im JS, Morrone A, Johung K, Goodwin EC, et al. Senescence-associated beta-galactosidase is lysosomal beta-galactosidase. *Aging Cell* 2006;**5**:187-195.
17. Sousa-Victor P, Gutarra S, García-Prat L, Rodriguez-Ubreva J, Ortet L, Ruiz-Bonilla V, et al. Geriatric muscle stem cells switch reversible quiescence into senescence. *Nature* 2014;**506**:316-321.
18. Uezumi A, Ito T, Morikawa D, Shimizu N, Yoneda T, Segawa M, et al. Fibrosis and adipogenesis originate from a common mesenchymal progenitor in skeletal muscle. *J Cell Sci* 2011;**124**:3654-3664.
19. Lee JE, Ge K. Transcriptional and epigenetic regulation of PPARγ expression during adipogenesis. *Cell Biosci* 2014;**4**:29.
20. Scicchitano BM, Dobrowolny G, Sica G, Musarò A. Molecular Insights into Muscle Homeostasis, Atrophy and Wasting. *Curr Genomics* 2018;**19**:356-369.
21. Tierney MT, Sacco A. Satellite Cell Heterogeneity in Skeletal Muscle Homeostasis. *Trends Cell Biol* 2016;**26**:434-444.
22. Brook MS, Wilkinson DJ, Phillips BE, Perez-Schindler J, Philp A, Smith K, et al. Skeletal muscle homeostasis and plasticity in youth and ageing: impact of nutrition and exercise. *Acta Physiol (Oxf)* 2016;**216**:15-41.
23. Moylan JS, Reid MB. Oxidative stress, chronic disease, and muscle wasting. *Muscle Nerve* 2007;**35**:411-429.
24. Faris R, Moore RA, Ward A, Race B, Dorward DW, Hollister JR, et al. Cellular prion protein is present in mitochondria of healthy mice. *Sci Rep* 2017;**7**:41556.

**Supplementary full length western blots image**

**Fig. 1d:**

**
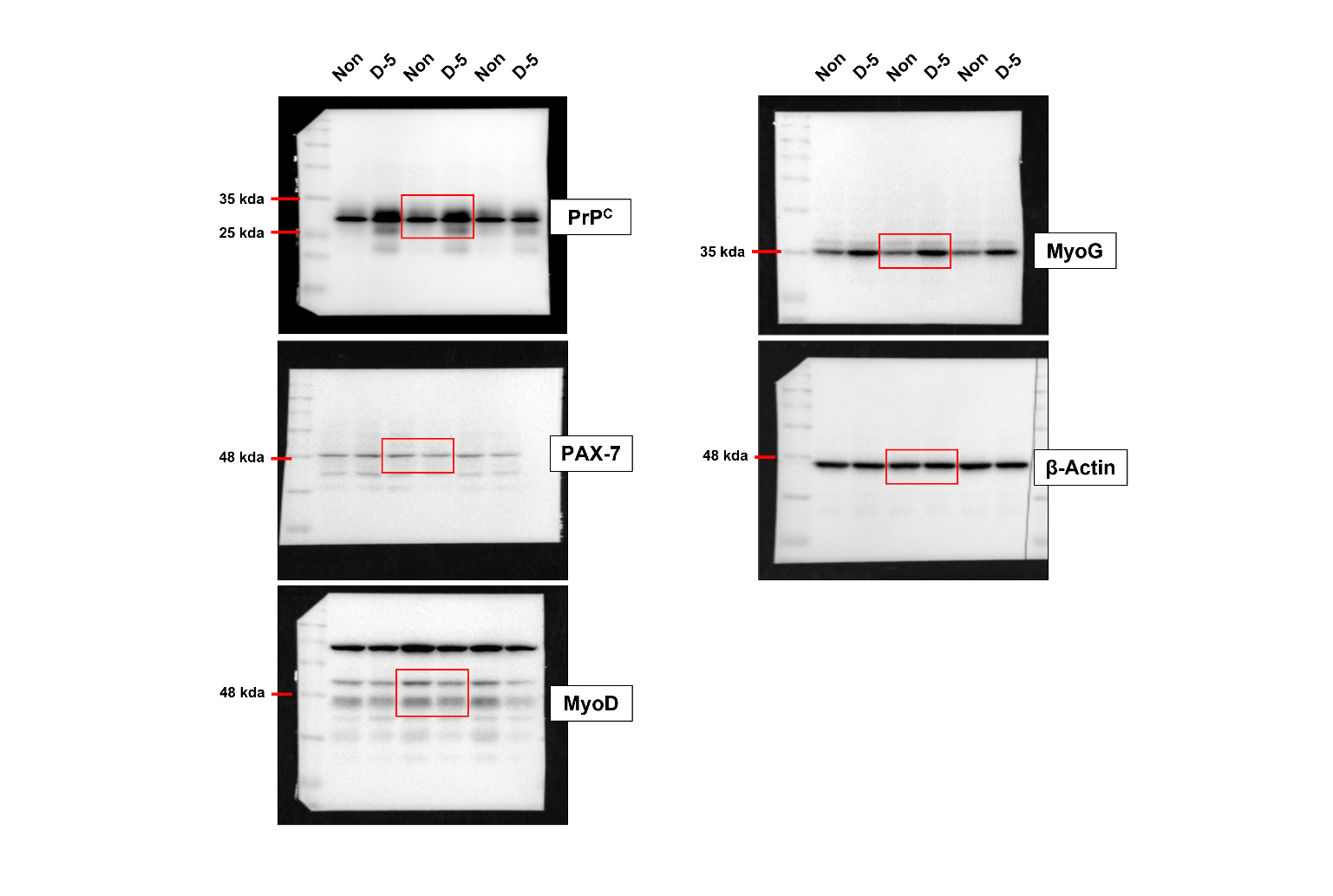
**

**Fig. 2b:**

**
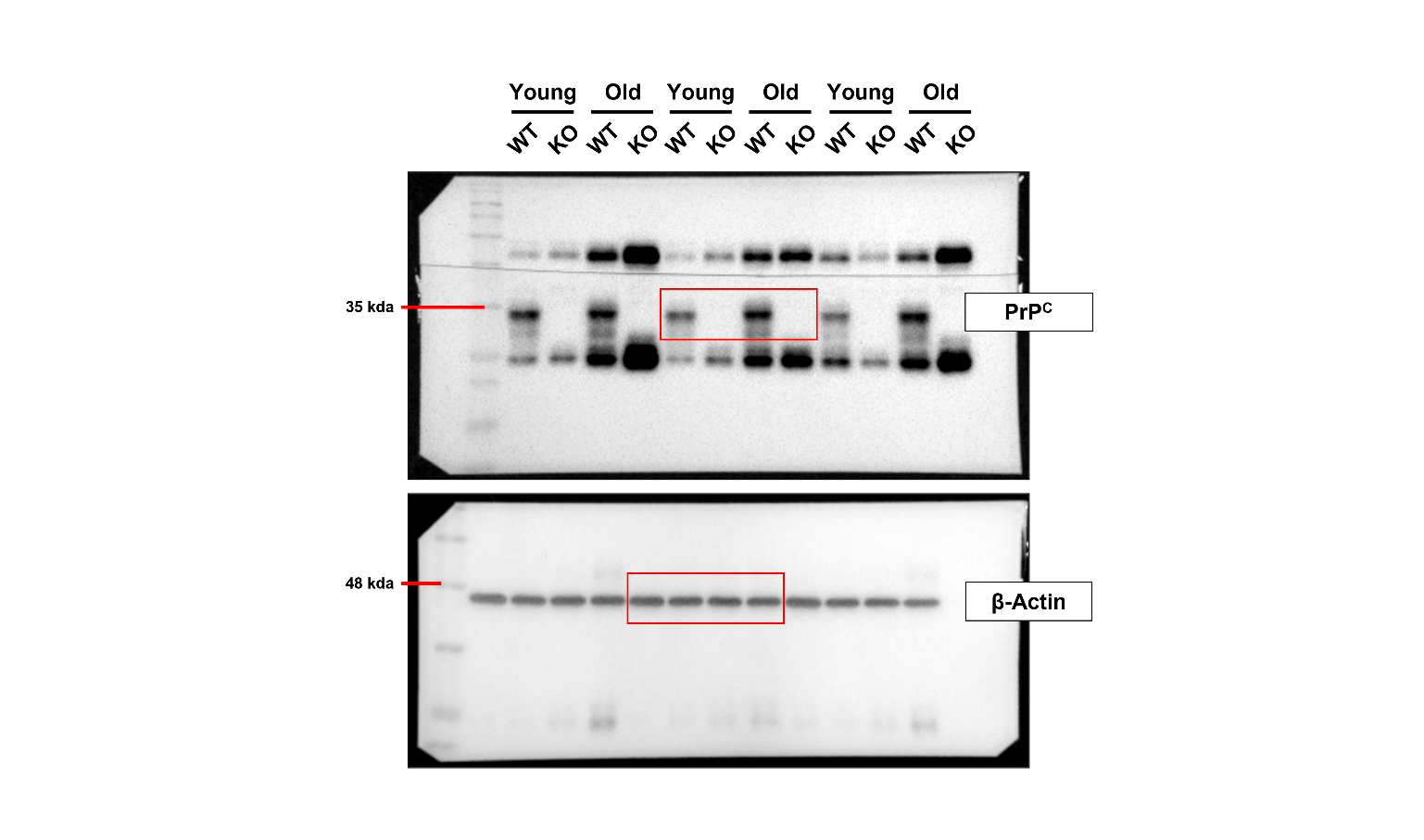
**

**Fig. 2e:**

**
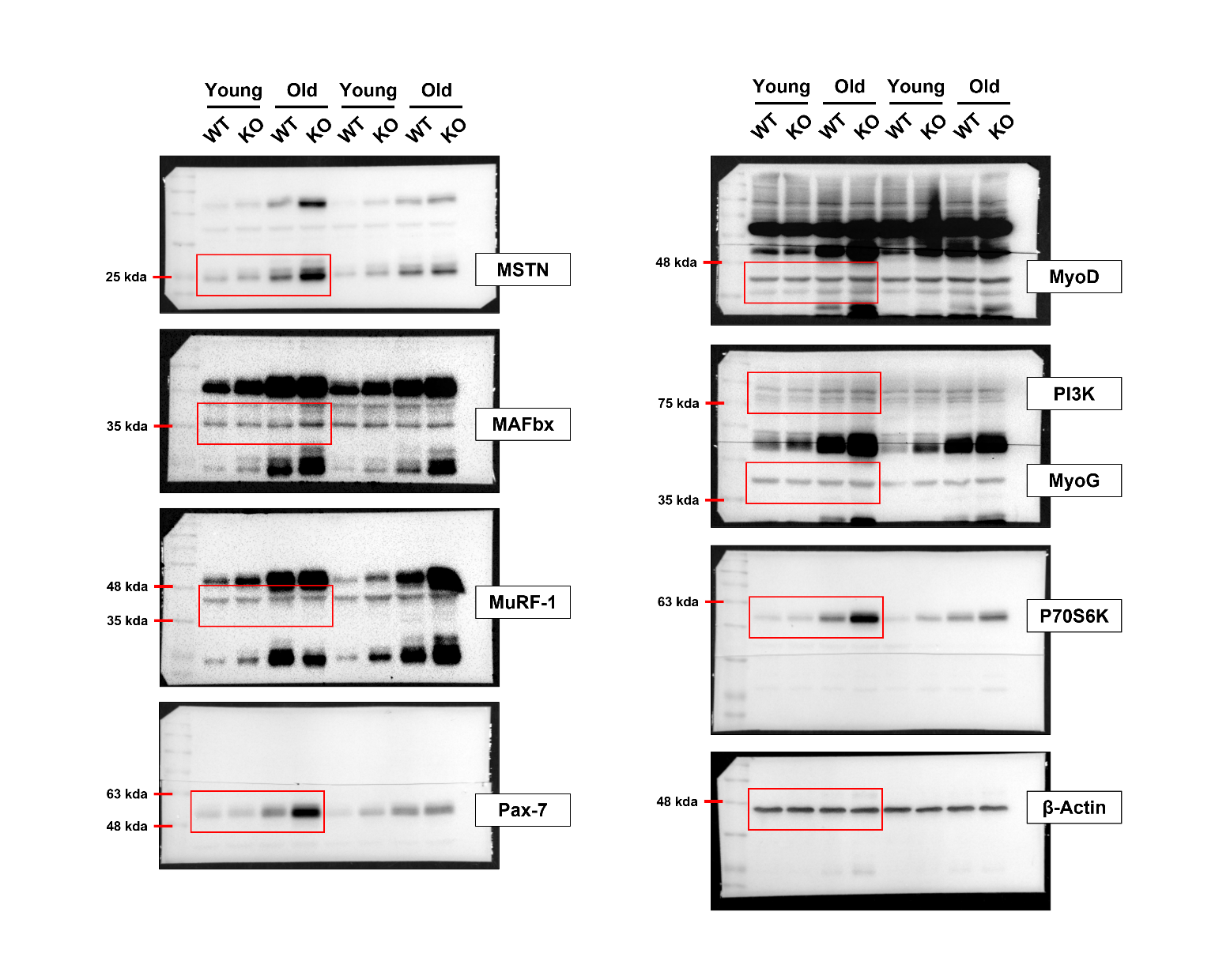
**

**Fig. 3c:**

**
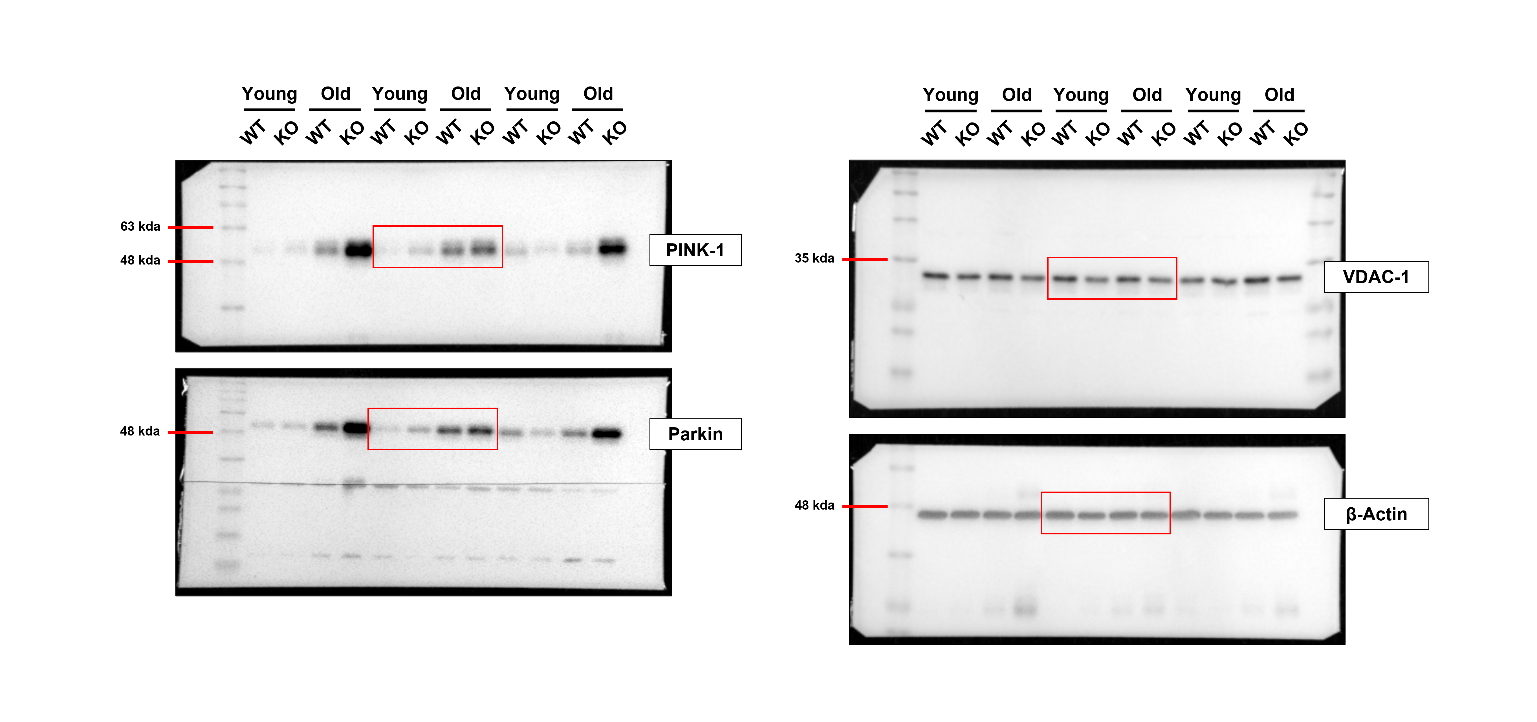
**

**Fig. 3d:**

**
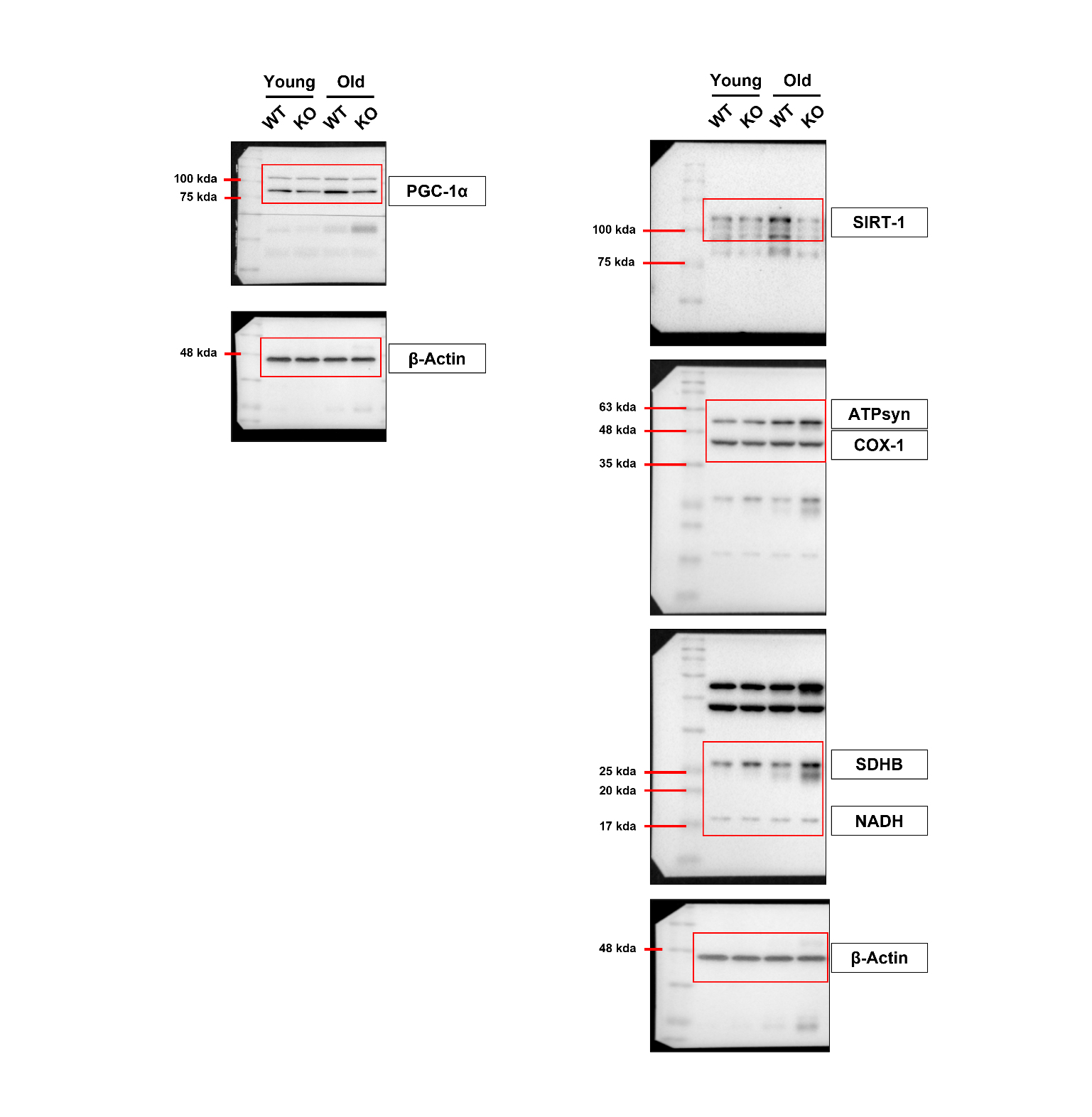
**

**Fig. 4c:**

**
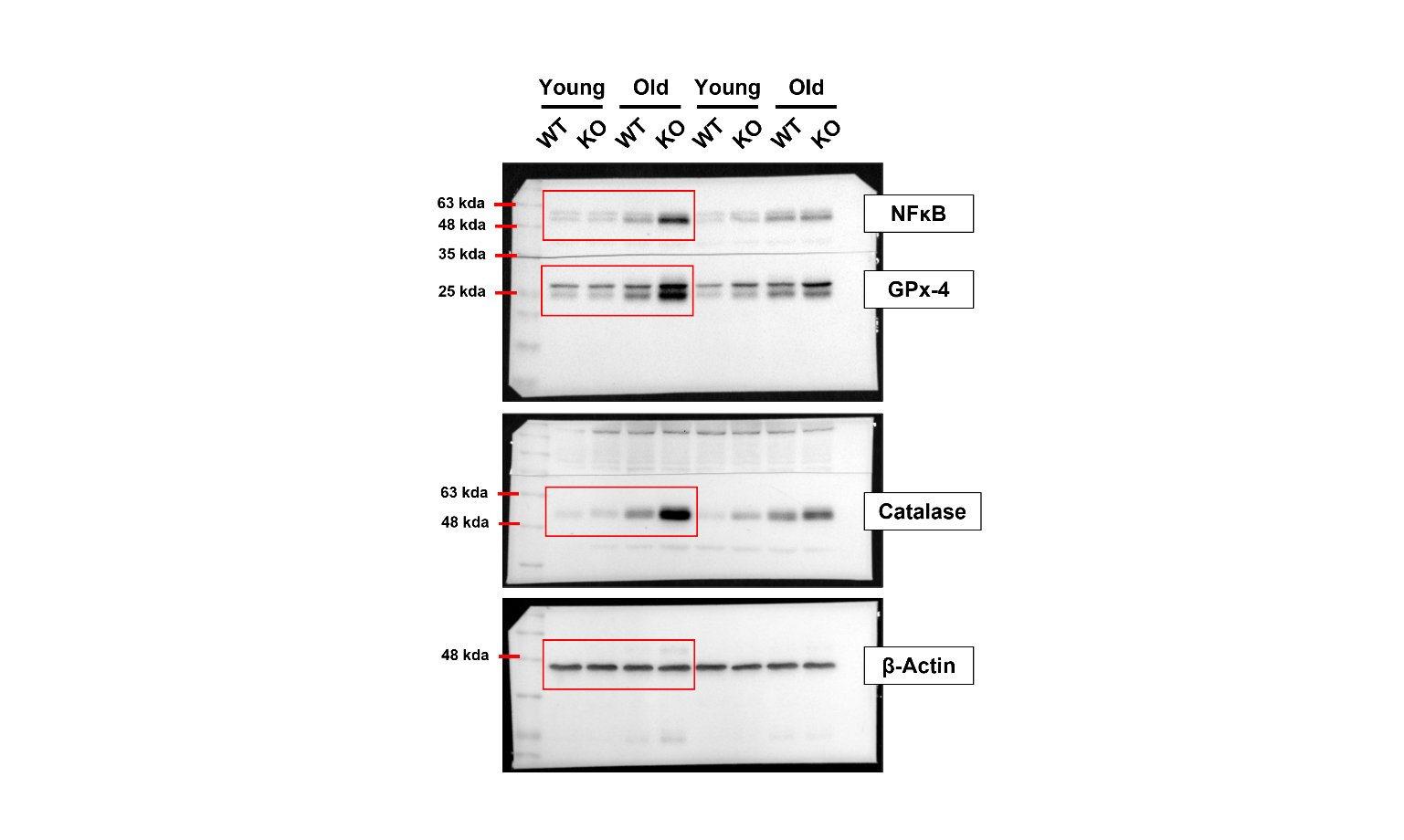
**

**Fig. 4d:**

**
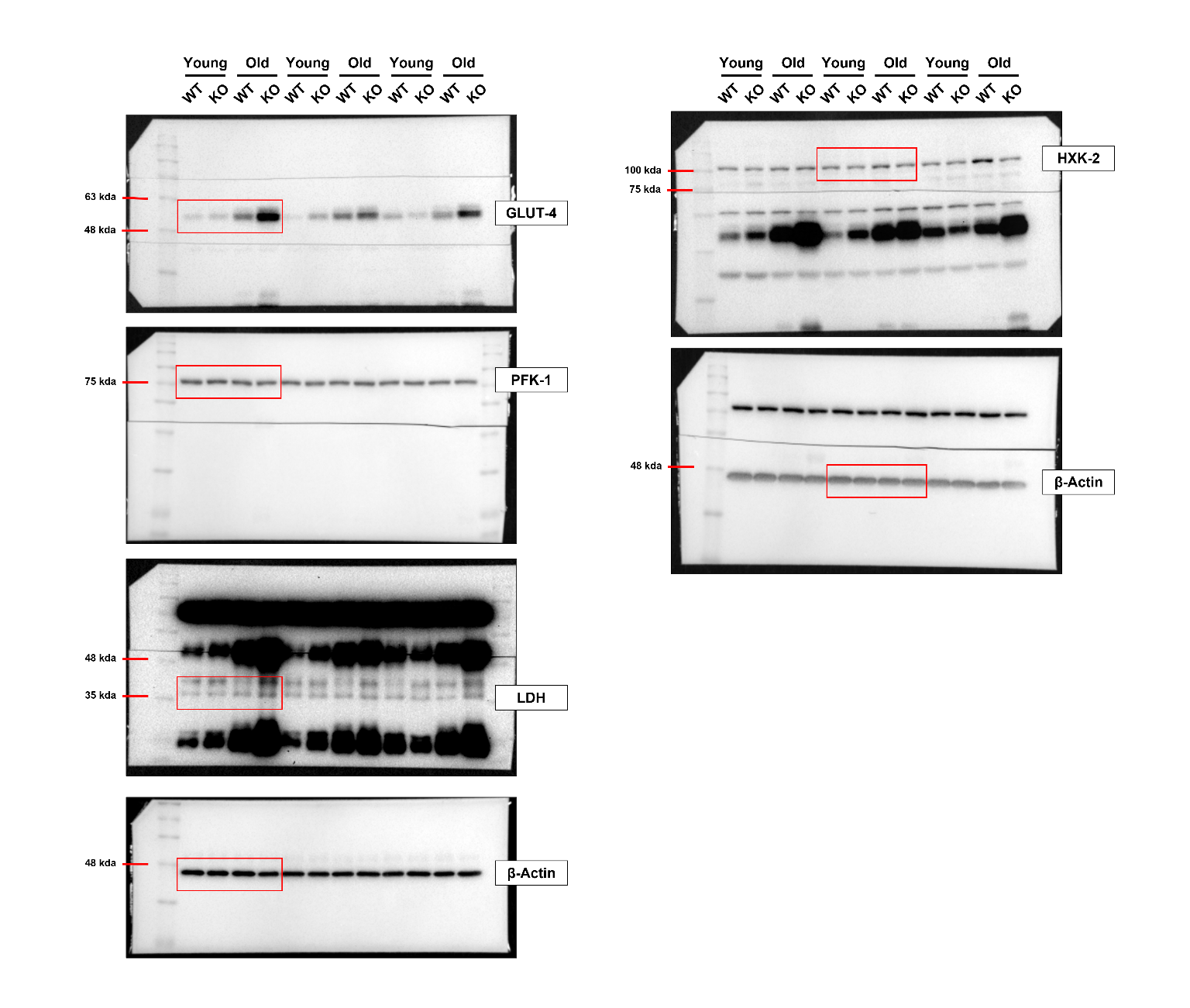
**

**Fig. 5c:**

**
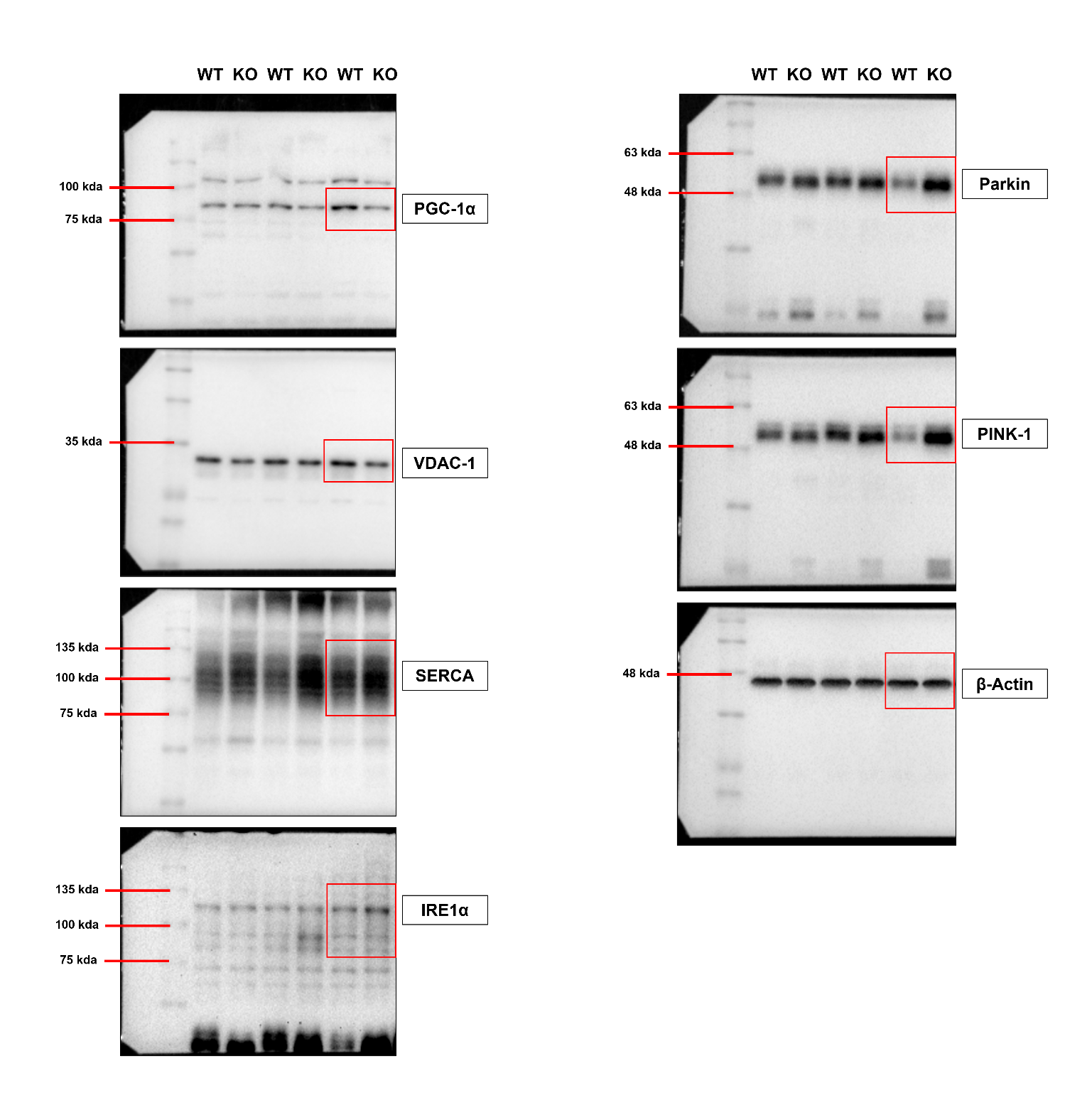
**

**Fig. 7e:**

**
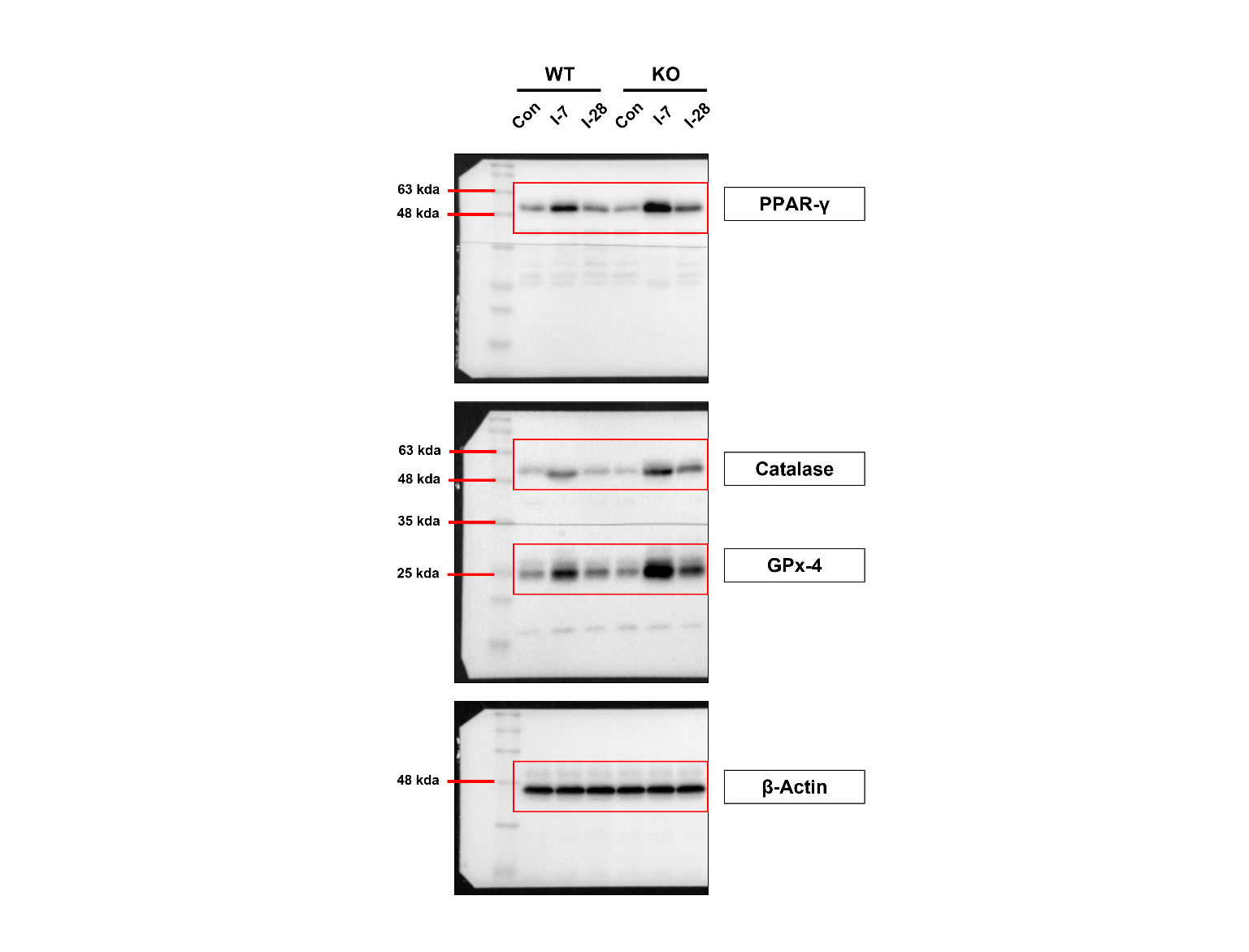
**

**Fig. S2:**

**
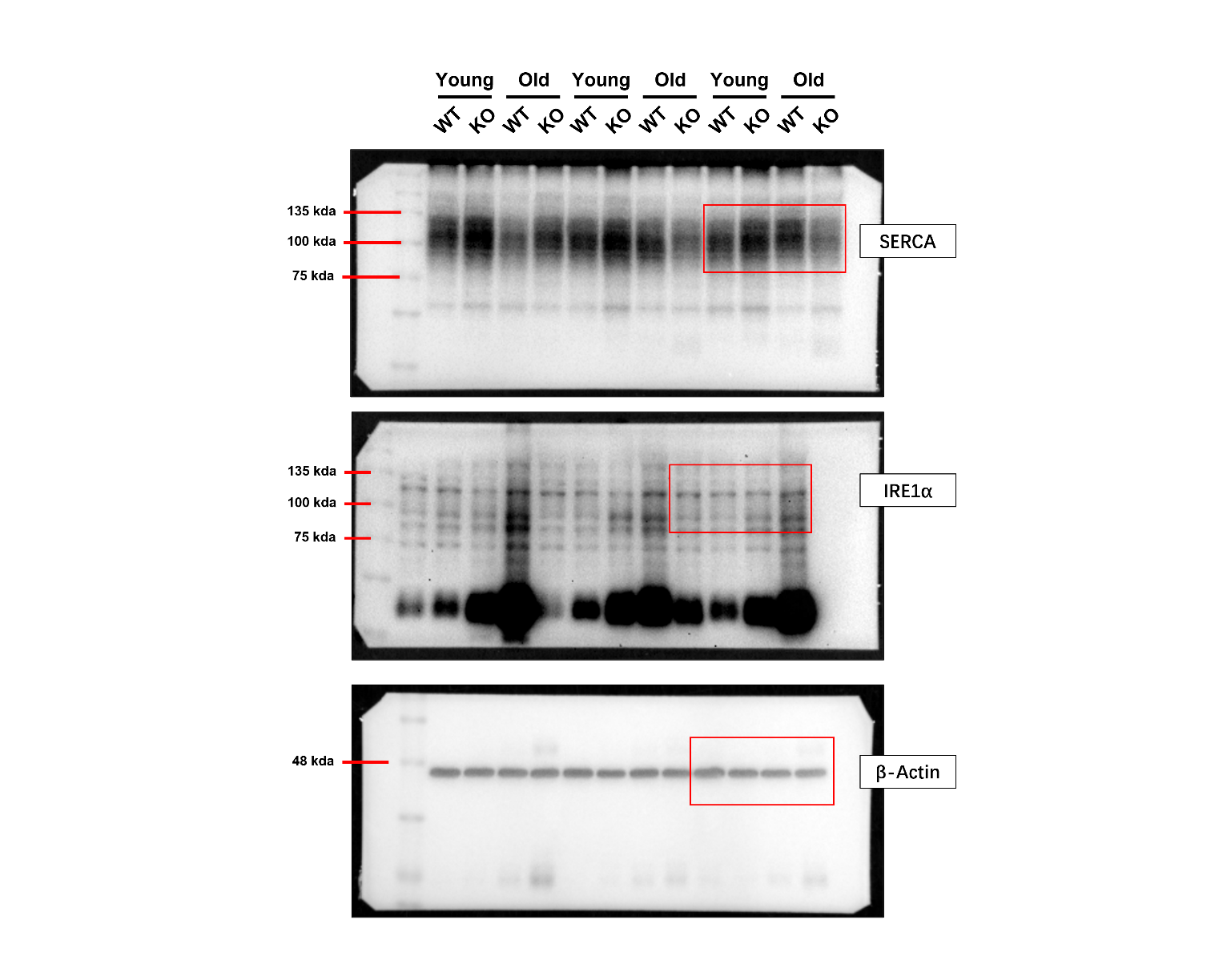
**

**Fig. S3:**

**
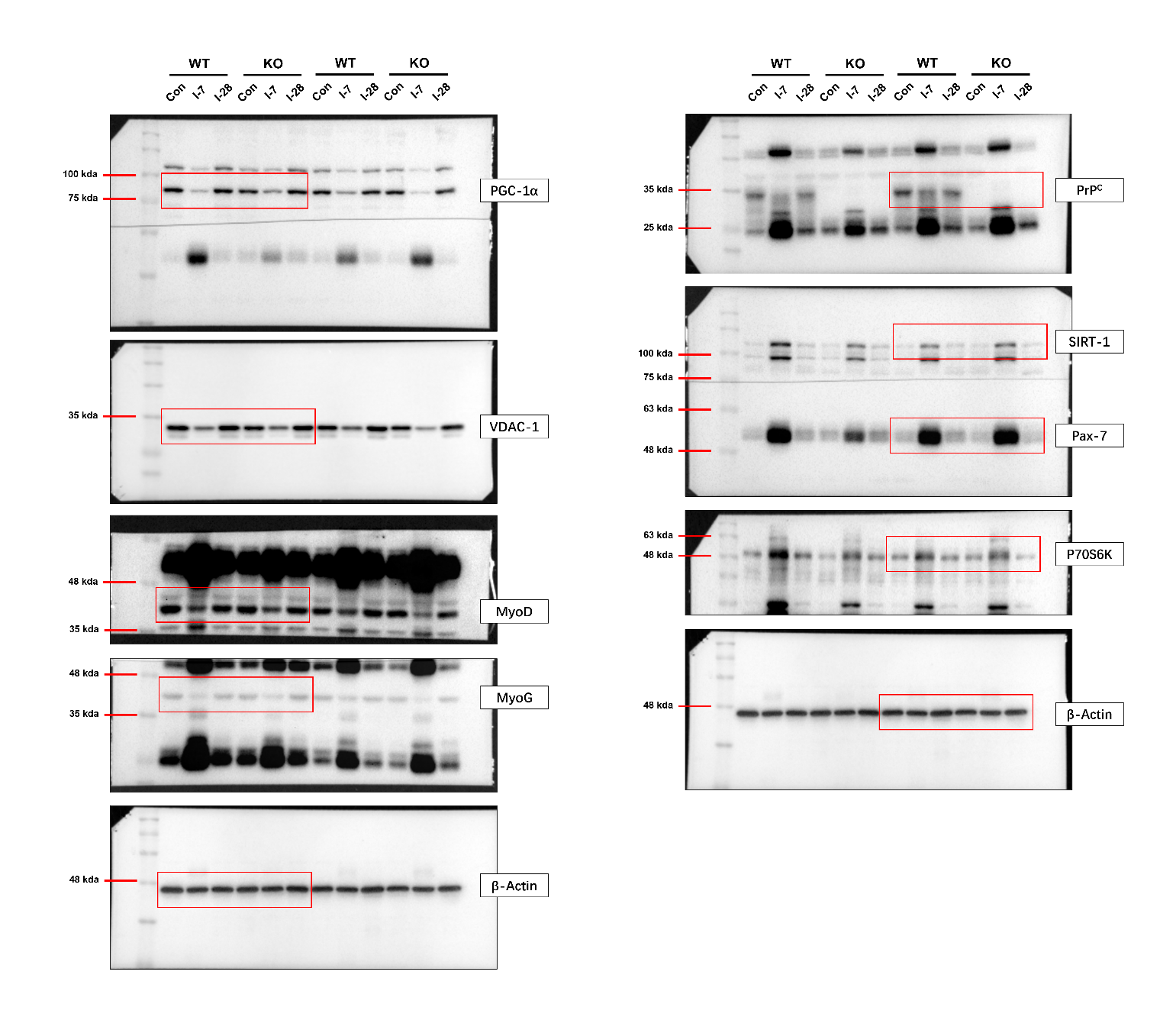
**
